# Whole tissue imaging of cellular boundaries at sub‐micron resolutions for deep learning cell segmentation: Applications in the analysis of epithelial bending of ectoderm

**DOI:** 10.1002/dvdy.70061

**Published:** 2025-07-26

**Authors:** Sam C. P. Norris, Jimmy K. Hu, Neil H. Shubin

**Affiliations:** ^1^ Department of Organismal Biology and Anatomy The University of Chicago Chicago Illinois USA; ^2^ School of Dentistry University of California Los Angeles Los Angeles California USA; ^3^ Molecular Biology Institute University of California Los Angeles Los Angeles California USA

**Keywords:** cell morphology quantification, deep learning in microscopy, dental developmental biology, developmental transitions, epithelial bending, mechanobiology, non‐model organisms, tissue clearing, whole tissue cell segmentation

## Abstract

**Background:**

To understand cellular morphology, biologists have relied on traditional optical microscopy of tissues combined with tissue clearing protocols to image structures deep within tissues. Unfortunately, these protocols often struggle to retain cell boundary markers, especially at high enough resolutions necessary for precise cell segmentation. This limitation affects the ability to study changes in cell shape during major developmental events.

**Results:**

We introduce a method that preserves cell boundary markers and matches the refractive index of tissues with water. This technique enables the use of high‐magnification, long working distance water‐dipping objectives that provide sub‐micron resolution images. We subsequently segment individual cells using a trained neural network segmentation model. These segmented images facilitate quantification of cell properties of the entire three‐dimensional tissue. As a demonstration, we examine mandibles of transgenic mice that express fluorescent proteins in their cell membranes and extend this technique to a non‐model animal, the catshark, investigating its dental lamina and dermal denticles—invaginating and evaginating ectodermal structures, respectively. This technique provides insight into the mechanical environment that cells experience during developmental transitions.

**Conclusions:**

This pipeline, named MORPHOVIEW, provides a powerful tool to quantify in high throughput the 3D structures of cells and tissues during organ morphogenesis.

## Key Findings

- Cell boundary markers for non‐model orgainisms.
- Refractive index matching of tissue to water.
- Whole tissue cell segmentation.
- Quantification of 3D cell shape properties.

## 1 INTRODUCTION

Tissue development is driven by precisely coordinated changes in cell shape, number, movement, and contractility over both time and space. To fully uncover the mechanisms driving morphogenesis, it is essential to better understand the native three‐dimensional (3D) properties of individual cells. Among these properties, cell shape is a particularly informative structural feature. For instance, when combined with computational modeling, cell shape can be used to quantify broader changes in tissue architecture,^1^ such as measuring apical constriction of cells to indicate local tissue bending.^2^ Cell shape can also be used to infer mechanical forces and physical constraints integral to tissue morphogenesis,^3^ where several mathematical models calculate mechanical stresses in tissues based on the shapes of their constituent cells. Furthermore, cell density and the cell shape index (a measure of how spherical or elongated a cell is) help identify transitions in tissue rigidity where cells “jam” and do not readily exchange neighbors.^4^, ^5^ In order to utilize cell shape to better understand morphogenesis, precise maps of 3D cell boundaries are required.

Biologists have long visualized the shape and internal structure of cells and tissues by imaging thin sections with light or electron microscopy. These two‐dimensional (2D) maps of biological information are analyzed to understand spatial relationships of RNA transcripts, proteins, cellular morphology, and tissue organization. Native tissue structures, however, are inherently 3D, justifying the need to move toward whole‐tissue microscopy. Scientists have used traditional approaches such as MRI, x‐ray computed tomography, and ultrasound imaging to acquire 3D information of tissues, but these techniques are unable to provide submicron resolutions, single molecule imaging, and lack a number of contrasting modalities compared to optical microscopy.^6^ Early methods to acquire 3D optical microscopy images include serial sectioning, but the resolution of the reconstructed 3D images is poor and limited by the slice thickness. Confocal and two‐photon microscopy of fluorescently tagged samples allow scientists to perform volumetric imaging at high resolutions,^7^ however, native tissues pose a complicated problem for optical microscopes: their composition of various lipids, proteins, minerals, pigments, among other components, renders the tissues optically opaque due to refractive index (RI) mismatches, thus scattering and distorting light, which limits the optical imaging depth of tissues.^8^

Efforts to increase the transparency of tissues have been ongoing for at least a century, where early attempts focused on matching the RI of the surrounding medium to the RI of the tissue, which was further improved by bleaching, decalcifying, dehydrating, and removing hydrophilic components from the tissues.^9^ In the past decade, the development of tissue clearing methods has accelerated: researchers have utilized a combination of advanced chemical techniques to remove high RI particles, such as lipids and fibrous proteins, endogenous pigments with strong absorption, such as heme and melanin, and the use of more efficient and appropriate RI‐matching chemical agents without damaging or altering the native cell and tissue structures of interest.^8^ The combination of tissue clearing, fluorescence labeling (small‐molecule dyes, tagged antibodies, and fluorescent proteins), and efficient confocal, two‐photon, and light‐sheet microscopes has revolutionized the ability of scientists to visualize biological specimens.^10^, ^11^, ^12^, ^13^

One particularly useful application of deep‐tissue volumetric imaging is the ability to assess 3D cellular and tissue morphology, which bypasses the need to section through different angles/axes to piece together tissue morphology. By segmenting individual cells, scientists can now quantify individual 3D cell properties such as volume, sphericity, and orientation instead of area, roundness, and other 2D cell properties obtained from often oblique tissue slices. In addition, 3D images also reveal morphological characteristics, such as protrusions and other cell–cell or cell–matrix interactions that would otherwise be lost. Recent advances in deep learning image processing techniques driven by a computer's graphics processing unit (GPU) have further improved our understanding of cell biology by accurately segmenting both individual nuclei and entire cell bodies in an automated fashion with little to no user input.^14^, ^15^ Such tools provide the ability to quantify the 3D morphology of thousands of cells at once.

To image cleared tissue samples and to automatically segment each individual cell within the tissues, several requirements must be met: (A) a high numerical aperture (N.A. ≥0.8), high magnification (≥25×) microscope objective is needed to obtain the necessary resolution to properly segment cell boundaries; (B) the objective must have a long working distance (WD), or enough to image through the entire tissue, ideally ≥1 mm; (C) the objective, tissue, and tissue mounting medium should all have the same RI to produce a homogeneous immersion system, where the RI of tissues is typically ≈1.45–1.5; (D) the cells must be stained with an appropriate marker that delineates cell boundaries. With the exception of a single Leica FLUOTAR objective (25×/1.0, WD 6 mm, multi‐immersion) there are currently no confocal objectives on the market that fulfill criteria A–C: higher power oil and glycerol objectives are limited to short WDs, typically <200 μm; and longer WD, high RI objectives are limited to lower magnification (<20×) and typically designed for light sheet microscopes. Finally, segmenting cell boundaries by either cytoplasmic or cell membrane markers can be challenging. While transgenic model species, such as mice and zebrafish, can be engineered to express genetically encoded membrane‐targeted fluorescent proteins for imaging, cell boundaries in wild‐type animals and non‐model species must be labeled exogenously. Unfortunately, lipophilic fluorescent dyes, such as DiI, and markers of filamentous actin (F‐actin), such as phalloidin, are incompatible with existing clearing methods as lipids and/or staining signals are lost during the clearing process.^16^

Here we describe a methodology termed MORPHOVIEW (Multispecies Optimized Refractive index matched tissue Preparation for High‐resolution Optical imaging, Visualization, Image segmentation, and Exploration of Whole tissues) that overcomes these limitations. First, since phalloidin is a consistent marker for cell body segmentation across various animal species, we devise a method to retain its signal during the clearing process. Next, we take advantage of high power, long WD, water dipping objectives commonly available from all the major confocal objective manufacturers (Zeiss, Leica, Nikon, Olympus) by digesting the tissues with Proteinase K, which renders them optically transparent in water. To prevent the enzymatic digestion from completely dissolving structures of interest, the antibodies and other fluorescent proteins are covalently bound into polyacrylamide network using a heterobifunctional linker.^17^ For proof of principle, we develop MORPHOVIEW using mouse and catshark embryos as model systems, focusing on oral dental lamina and dermal denticle structures. To further demonstrate the applicability of this technique to a broad phylogenetic range, we apply MORPHOVIEW to xenopus tadpoles without any modification to the protocol. Our results show that the technique allows the segmentation of every individual cell in the tissues of interest and quantify their 3D morphological characteristics with both speed and precision.

## 2 RESULTS AND DISCUSSION

### 2.1 RI matching solutions improve optical penetration depth but disrupt fluorescent phalloidin labeling

To fully appreciate how tissues develop, it is desirable to fully characterize individual cell morphologies in their native 3D form. Cell body segmentation, however, requires a detectable marker that delineates cell boundaries; in mice, this is generally achieved using genetically encoded fluorescent proteins that label the cell membrane or nucleus. For example, *K14* ^*Cre*^;*R26* ^*mT/mG*^ mice express a *Keratin 14* promoter‐driven Cre recombinase that activates the expression of membrane GFP in ectodermally derived epithelial tissues, such as the developing dental epithelium. In mesenchymal cells where the Cre is inactive, membrane tdTomato is not recombined out and continues to be expressed. These fluorescent signals can thus be used to delineate the membrane boundary of individual epithelial (GFP‐positive) and mesenchymal cells (tdTomato‐positive).

**FIGURE 1:**
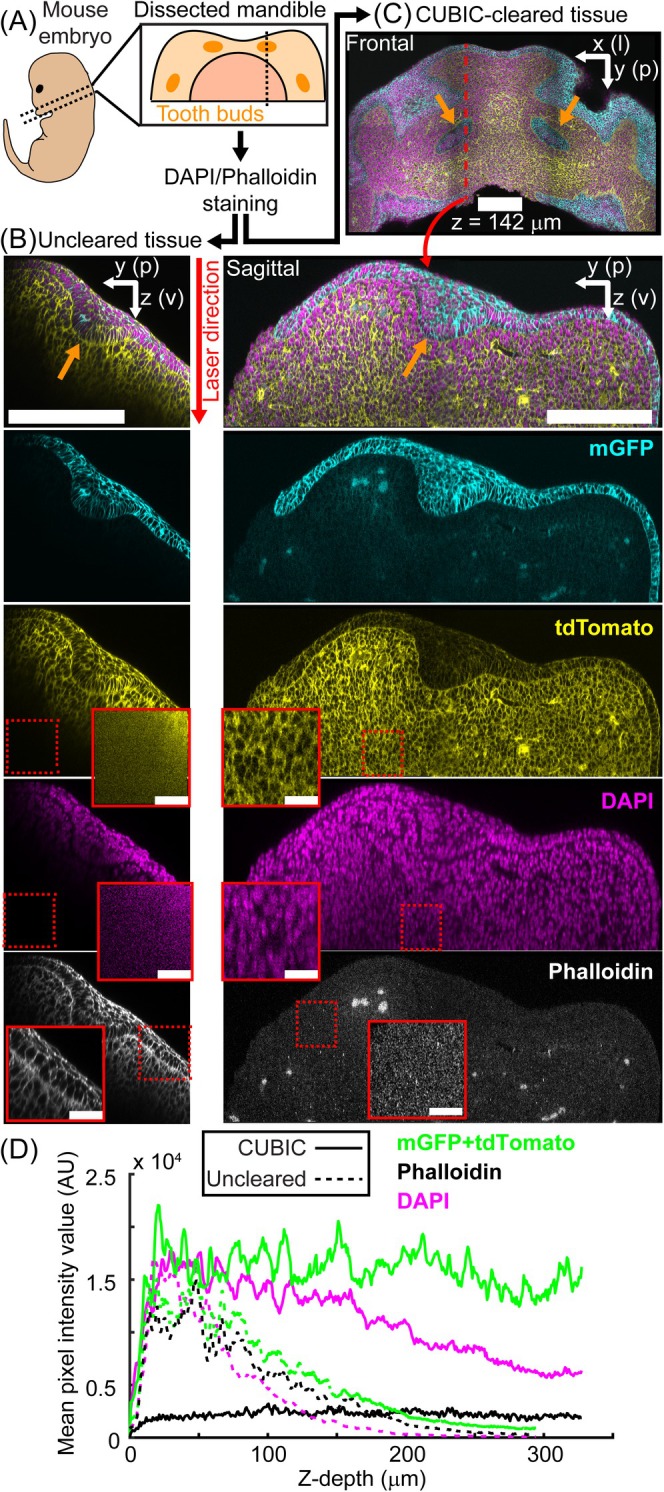
RI‐matching improves deep‐tissue imaging of genetically encoded fluorescent proteins. (A) E12.5 *K14* ^*Cre*^;*R26* ^*mT/mG*^ mouse mandibles were dissected and counterstained with DAPI and phalloidin‐Alexa Fluor 633. Epithelial cells are labeled by membrane GFP (mGFP), mesenchymal cells retain expression of membrane tdTomato, while all cells are labeled by phalloidin and DAPI. The mandibles were then either imaged directly, or after incubation in the RI‐matching solution CUBIC‐R. (B) Mouse mandible without any tissue clearing. 3D confocal volume imaged using a 40×/1.0 water dipping objective. Image shows a virtual sagittal (YZ) section of the mouse mandibular incisor tooth bud. Individual channels and contrast‐adjusted zoom‐in inset images are shown. Orange arrows indicate the incisor tooth bud. (C) Mouse mandible cleared with CUBIC‐R RI‐matching media. 3D confocal volume acquired using a 25×/0.8 glycerol immersion objective. Both virtual frontal (XY) and sagittal (YZ) sections are shown with individual channels and contrast‐adjusted zoom‐in inset images shown for the sagittal section. Frontal section taken at a depth of 142 μm. Dashed red line of the frontal section indicates the location of the sagittal plane through the tooth bud. Orange arrows indicate the incisor tooth bud. (D) Image intensity profile plot as a function of laser penetration depth (Z‐direction) of the confocal volumes. Comparison is made between CUBIC‐R cleared (solid lines) and uncleared (dashed lines) tissues. Since mGFP is specific to the epithelium, and tdTomato the mesenchyme, the two channels were added together before analysis. Profile plots of the DAPI and phalloidin signals are also shown. Scalebars: (B,C) 200 μm; all zoomed‐in insets: 30 μm.

A key challenge in visualizing cell boundaries deep into tissue is the inherent tissue opacity. For instance, when embryonic day (E) 12.5 *K14* ^*Cre*^;*R26* ^*mT/mG*^ mouse mandibles, counterstained with DAPI and phalloidin (Figure 1A), were imaged with a confocal microscope using a 40×/1.0 water dipping objective, the entire incisor tooth bud was visible, but the resolution of cellular structures rapidly diminished beyond ~100 μm (Figure 1B). To overcome this limitation, we incubated the mandibles in a RI‐matching solution, CUBIC‐R,^18^ which rendered the tissue optically transparent, and imaged them with a 25×/0.8 glycerol immersion objective. These images demonstrate clear visualization of cellular boundaries and tissue structures deep into the mesenchyme, beyond the incisor tooth bud in the DAPI, membrane GFP, and membrane tdTomato signals (Figure 1C and Video 1). To quantify improvements in imaging depth, the signal intensities were plotted as a function of laser penetration depth (Figure 1D). RI‐matching with CUBIC‐R improved tissue transparency and significantly reduced signal attenuation in the GFP and tdTomato channels, with little‐to‐no signal loss observed. For example, the tdTomato signal was undetectable beyond ~100 μm in uncleared tissues (Figure 1B tdTomato zoom‐in), but was clearly retained in CUBIC‐R‐cleared tissues (Figure 1C tdTomato zoom‐in). In the DAPI channel, which utilizes a shorter wavelength, 405 nm laser, tissue clearing substantially increased signal penetration depth, although moderate attenuation remained (Figure 1D). Nonetheless, DAPI‐labeled nuclei remained distinct and readily identifiable in cleared tissues (Figure 1B,C DAPI zoom‐in).

**VIDEO 1:** 3D Confocal volume of E12.5 *K14* ^*Cre*^;*R26* ^*mT/mG*^ mouse mandible cleared with CUBIC‐R. Cells are labeled by membrane GFP (mGFP, cyan), mesenchymal cells retain expression of membrane tdTomato (yellow). Animation shows the orthogonal planes of the volume.

**FIGURE 2:**
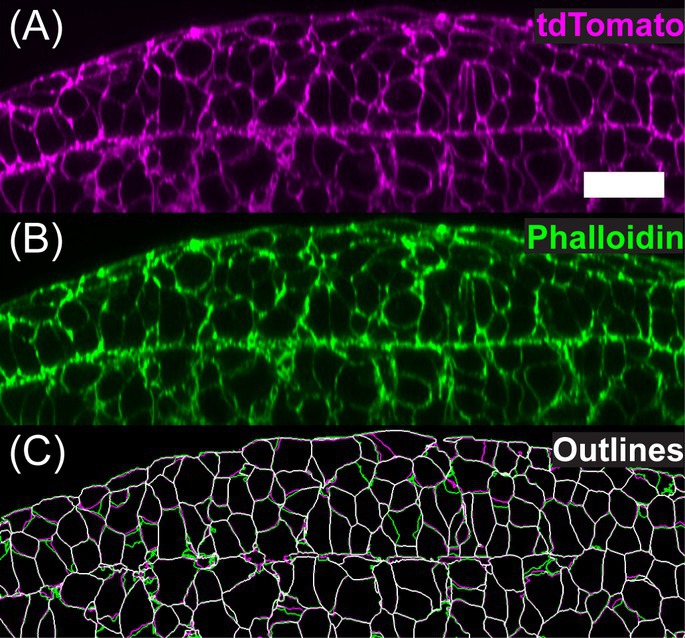
Phalloidin as a cell boundary marker. Example virtual XZ‐plane section of a 3D confocal volume of a E11.5 *K14* ^*Cre*^;*R26* ^*mT/mG*^ mouse mandible counterstained with phalloidin‐Alexa Fluor 633 imaged using a 40×/1.0 water immersion objective. No optical clearing agents were used. Image shows a sagittal view of mouse mandibular incisor tooth bud. (A) Expression of membrane tdTomato and (B) dye‐conjugated phalloidin. (C) Computer‐generated cell boundaries segmented using CellPose software from the tdTomato (magenta lines) and phalloidin (green lines) signals. White lines indicate overlap of the cell segmentations from the two different signal types. Scalebar: 25 μm.

While signals from genetically encoded fluorescence (membrane GFP and tdTomato) and DAPI were preserved after RI‐matching, phalloidin staining of F‐actin was lost (Figure 1C,D). Ultimately, we were unable to find any established RI‐matching agents that did not disrupt phalloidin binding. This deficit is unfortunate since actin is highly conserved, phalloidin exhibits broad species reactivity,^19^ and serves as a good marker of cell boundaries (Figure 1B). We also observed strong colocalization between tdTomato and phalloidin signals in E11.5 *K14* ^*Cre*^;*R26* ^*mT/mG*^ mouse mandibles (Figure 2A,B), and cell segmentation results based on these two markers produced highly similar cell boundary delineations (Figure 2C).

### 2.2 Fluorescent phalloidin labeling is maintained after tissue clearing using anti‐fluorophore antibodies

As proof‐of‐principle and to provide a morphologically and phylogenetically divergent comparison to the embryonic mouse, we utilized catshark embryos (Figure 4A), a non‐traditional model species, to perform whole‐tissue imaging of cellular boundaries. Our goal was to develop a protocol applicable to a wide variety of animal models. For wild‐type or non‐traditional model animals that do not express fluorescent proteins labeling cell membranes, we sought alternative methods to fluorescently tag cell boundaries.

**FIGURE 3:**
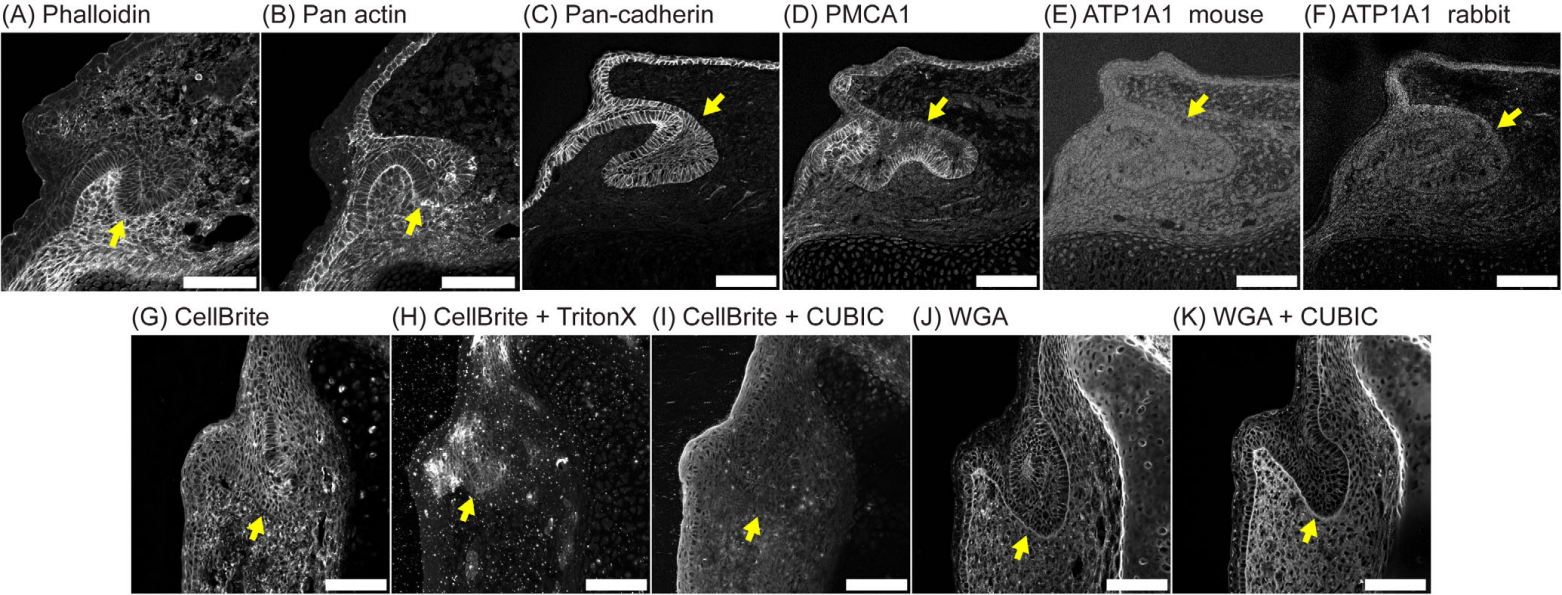
Compatibility of traditional cell boundaries markers. Representative serial thin sections of stage 33 catshark mandibles showing the dental lamina (yellow arrows) labeled with: (A) phalloidin; and potential markers for cell boundaries using (B–F) antibodies; and (G–K) non‐antibody‐based cell membrane markers. (B) Anti‐pan actin, (C) anti‐pan cadherin intercellular junction marker, (D) anti‐plasma membrane Ca^2+^ ATPase 1 (PMCA1), (E) mouse anti‐alpha 1 sodium potassium ATPase (ATP1A1), (F) rabbit ATP1A1, (G) CellBrite, (H) CellBrite then permeabilized with 0.1% Triton‐X, (I) CellBrite then mounted with CUBIC‐R, (J) wheat germ agglutinin (WGA), (K) WGA then mounted with CUBIC‐R. Scalebars 100 μm.

Given that phalloidin proved to be an effective marker for cell boundaries in mice, and considering the broad compatibility of tissue clearing protocols with antibody staining, we evaluated the efficacy of anti‐pan actin antibodies for labeling cell boundaries. While the anti‐pan actin antibodies were reactive with catshark tissues, they did not label cell boundaries as effectively as phalloidin (Figure 3A,B). Specifically, the antibodies failed to adequately label apical epithelial cells and certain mesenchymal cells. We next tested a panel of potential plasma membrane markers, including plasma membrane calcium‐transporting ATPase 1 (PMCA1); sodium potassium ATPase (Na/K ATPase); and pan cadherin (Figure 3C–F). Although both anti‐PMCA1 (Figure 3D) and pan‐cadherin (Figure 3C) antibodies were reactive with catshark tissues, they only successfully labeled epithelial cell boundaries and not the mesenchyme. Sections stained with either mouse (Figure 3E) or rabbit (Figure 3F) anti‐Na/K ATPase antibodies showed no specific binding in either epithelial or mesenchymal cells.

Next, we examined non‐antibody‐based cell membrane markers. Wheat germ agglutinin (WGA), a lectin that binds to glycoconjugates, when conjugated to fluorescent dyes is used to label cell membranes. Additionally, lipophilic dyes, such as DiI and its derivatives, are also used to stain lipid bilayers. Thin sections of catshark mandibles were stained with WGA or the DiI derivative, CellBrite, with phalloidin stained sections used as a control (Figure 3G–K). CellBrite successfully labeled cell boundaries (Figure 3G); however, its labeling was severely disrupted when exposed to 0.1% Triton‐X (Figure 3H) or CUBIC‐R (Figure 3I). A notable advantage of WGA, a protein, is that it contains primary amines and can be crosslinked into place using formaldehyde, ensuring signal stability during optical clearing (Figure 3J,K). We found, however, that WGA acted more as a broad stain for non‐nuclear cell matter, and its labeling of cell boundaries was less precise compared to phalloidin.

**FIGURE 4:**
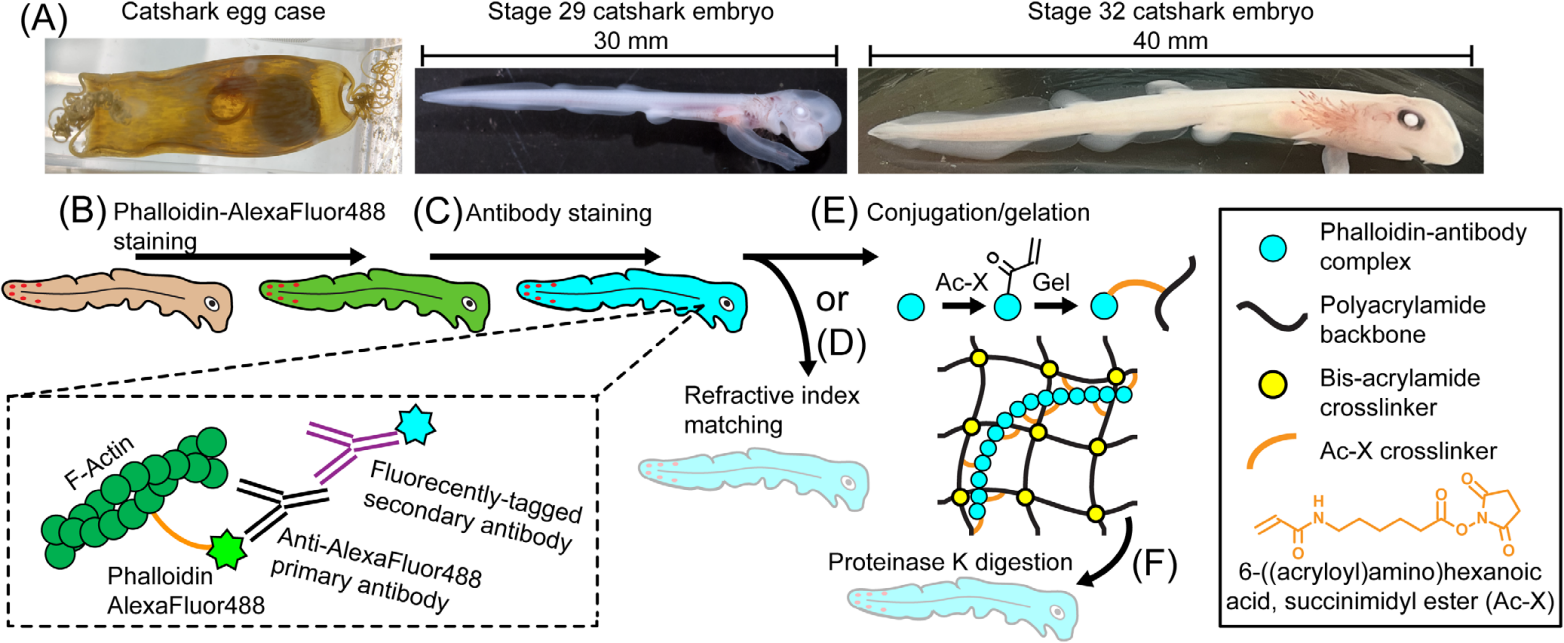
Schematic of the MORPHOVIEW workflow using catshark embryos as an example. (A) Images of the catshark embryo in its egg case, and stage 29 and stage 32 embryos after removal from the egg case. (B) After fixation, permeabilization, and blocking, tissues are stained with phalloidin conjugated to Alexa Fluor 488. (C) Phalloidin is then antibody‐tagged with anti‐fluorescent dye (Alexa Fluor 488) primary antibodies, followed by fluorescently‐tagged secondary antibodies. (D) At this point, the sample can undergo RI‐matching and imaging. (E) Alternatively, tissues can be incubated in a heterobifunctional linker (Ac‐X) that converts primary amines on the antibodies to reactive acrylamide groups. These acrylamide groups are then polymerized along with acrylamide monomers and bis‐acrylamide crosslinkers into a polyacrylamide hydrogel network. (F) From here, the tissue can be digested with Proteinase K to RI‐match the tissue to water and imaged with a water immersion objective.

**FIGURE 5:**
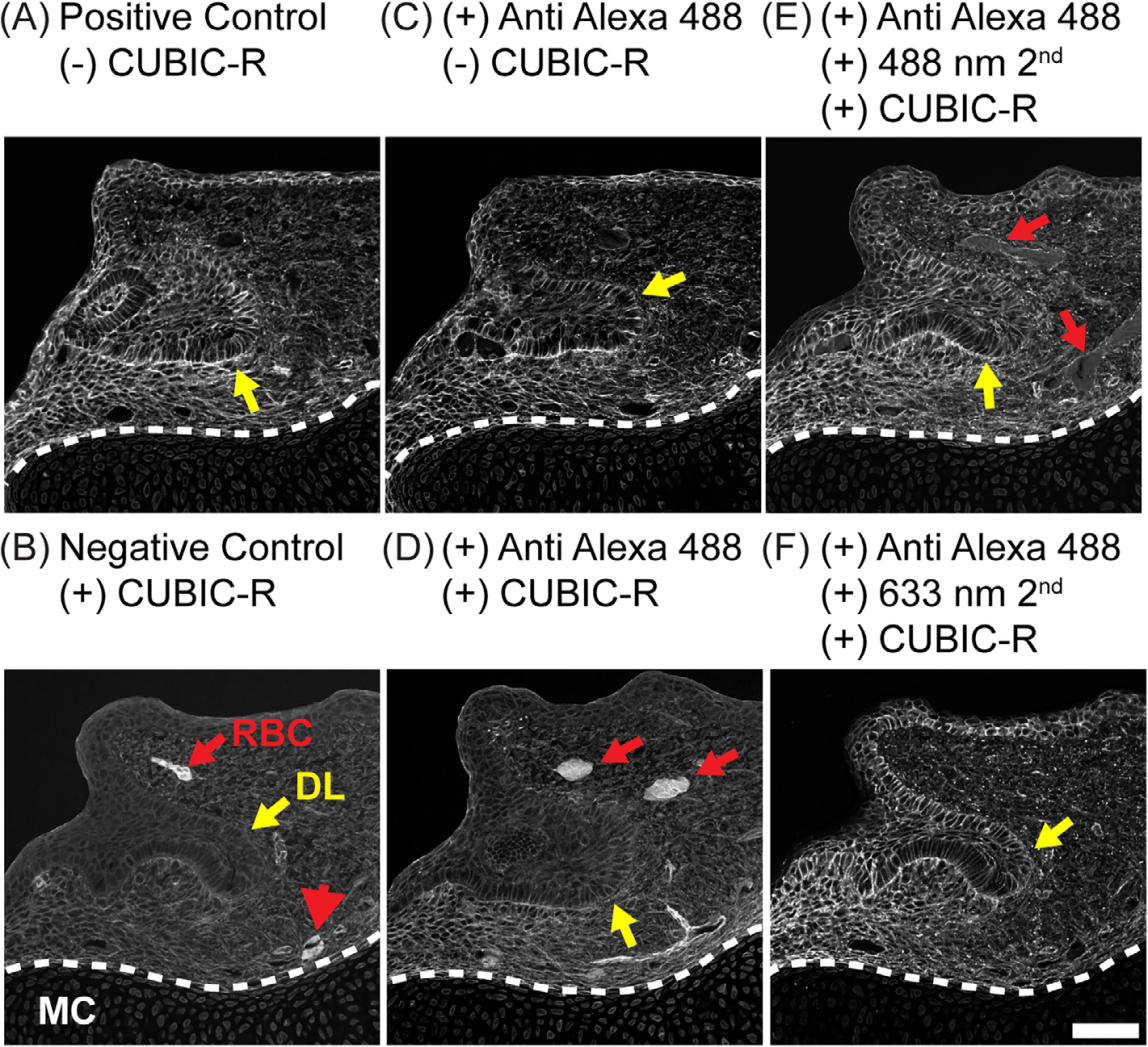
Phalloidin signal retention. Representative serial thin sections of stage 33 catshark mandibles showing the dental lamina (DL, yellow arrows) and labeled with phalloidin‐Alexa Fluor 488. Red arrows indicate red blood cells (RBC), and the boundary of the Meckel's cartilage (MC) is outlined with a dashed line. Sections without antibodies mounted in (A) PBS or (B) CUBIC‐R. Sections incubated in anti‐Alexa Fluor 488 primary antibodies mounted in (C) PBS or (D) CUBIC‐R. Sections incubated in anti‐Alexa Fluor 488 primary antibodies and either (E) Alexa Fluor 488 or (F) CF633 secondary antibodies then mounted in CUBIC‐R. Scalebar: 100 μm.

Given that phalloidin labeling was identified to be an accurate marker for cell boundaries (Figure 2), but the signal was lost after RI‐matching with CUBIC‐R (Figures 1C,D and 5A,B), we sought methods to retain the phalloidin signal following optical tissue clearing. Inspired by previously reported techniques that stabilize phalloidin binding,^16^ we developed a method to preserve phalloidin as a cell boundary marker for high‐resolution whole‐tissue imaging (Figure 4). In our approach, phalloidin‐Alexa Fluor 488 was labeled with an anti‐Alexa Fluor 488 antibody (Figures 4B,C and 5C,D). After a post‐antibody labeling fixation step, the tissues were incubated in a RI‐matching solution (Figure 4D), allowing for optical clearing without disrupting phalloidin binding patterns (Figure 5E,F). Additionally, we confirmed that anti‐Alexa Fluor 488 alone did not interfere with phalloidin binding (Figure 5A,C). Although Alexa Fluor 488 secondary antibodies significantly enhanced the phalloidin signal (Figure 5E), strong autofluorescence of red blood cells was observed in the 488 nm channel but not in the 633 nm channel when far‐red secondary antibodies were used (Figure 5F). The use of far‐red secondary antibodies also allowed us to image the DAPI channel simultaneously since DAPI is sufficiently spectrally separated from the far‐red fluorescent dyes. When applied to whole‐mount embryonic catshark tissues, this technique preserved the phalloidin signal after RI‐matching, enabling imaging at sub‐micron resolutions at full tissue penetration using confocal microscopy (Figure 7A).

### 2.3 Whole tissue imaging is accomplished using proteolytic digestion and long WD water dipping objectives

**FIGURE 6:**
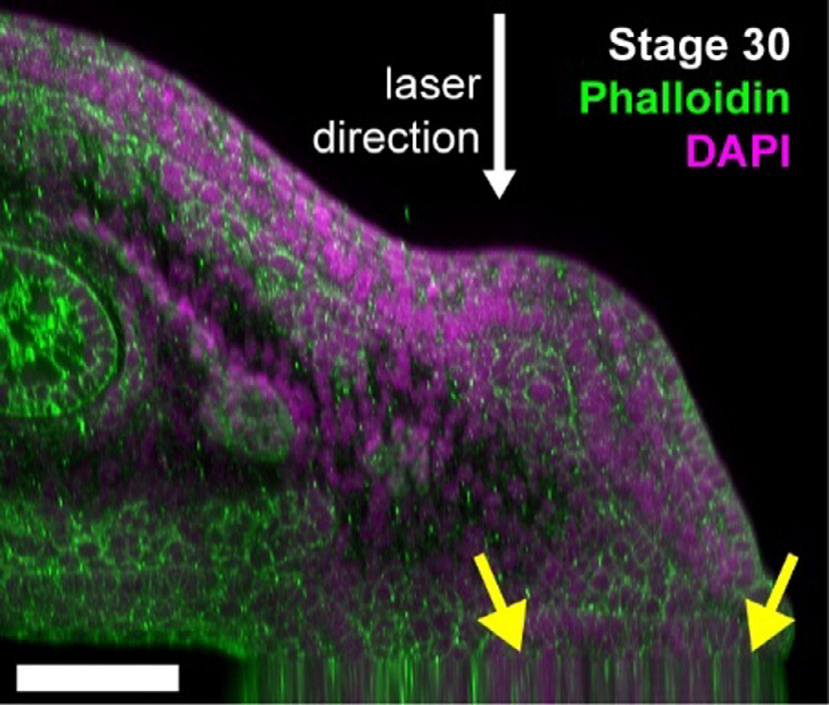
Limited working distance of high‐RI objectives. Example virtual section from 3D confocal volume of a stage 30 catshark tail tip tissue with phalloidin retention protocol, counterstained with DAPI, and optically cleared with CUBIC‐R RI matching media. Yellow arrows indicate region where the objective has bottomed out and cannot image tissue at deeper penetration depths. Scalebar: 100 μm.

A major limitation of the protocol outlined in Figure 4D is that high powered (>25×) glycerin or oil immersion objectives are manufactured with limited WDs (<600 μm for all Zeiss, Olympus, Leica, and Nikon objectives). This restricts full‐thickness imaging of larger tissue samples (Figure 6). To address this limitation, we sought a method enabling the use of high‐magnification water‐immersion objectives, which provide longer WDs (>1 mm) and are widely available across major confocal objective manufacturers. Consequently, we adapted the protein‐retention Expansion Microscopy (proExM) technique.^11^, ^17^, ^19^ After antibody labeling, we conjugated the tissue proteins and antibodies to a polyacrylamide hydrogel (Figure 4E) and subjected the tissue‐gel construct to nonspecific proteolytic digestion. This process rendered the samples transparent in water and compatible with water immersion objectives (Figure 4F).

**FIGURE 7:**
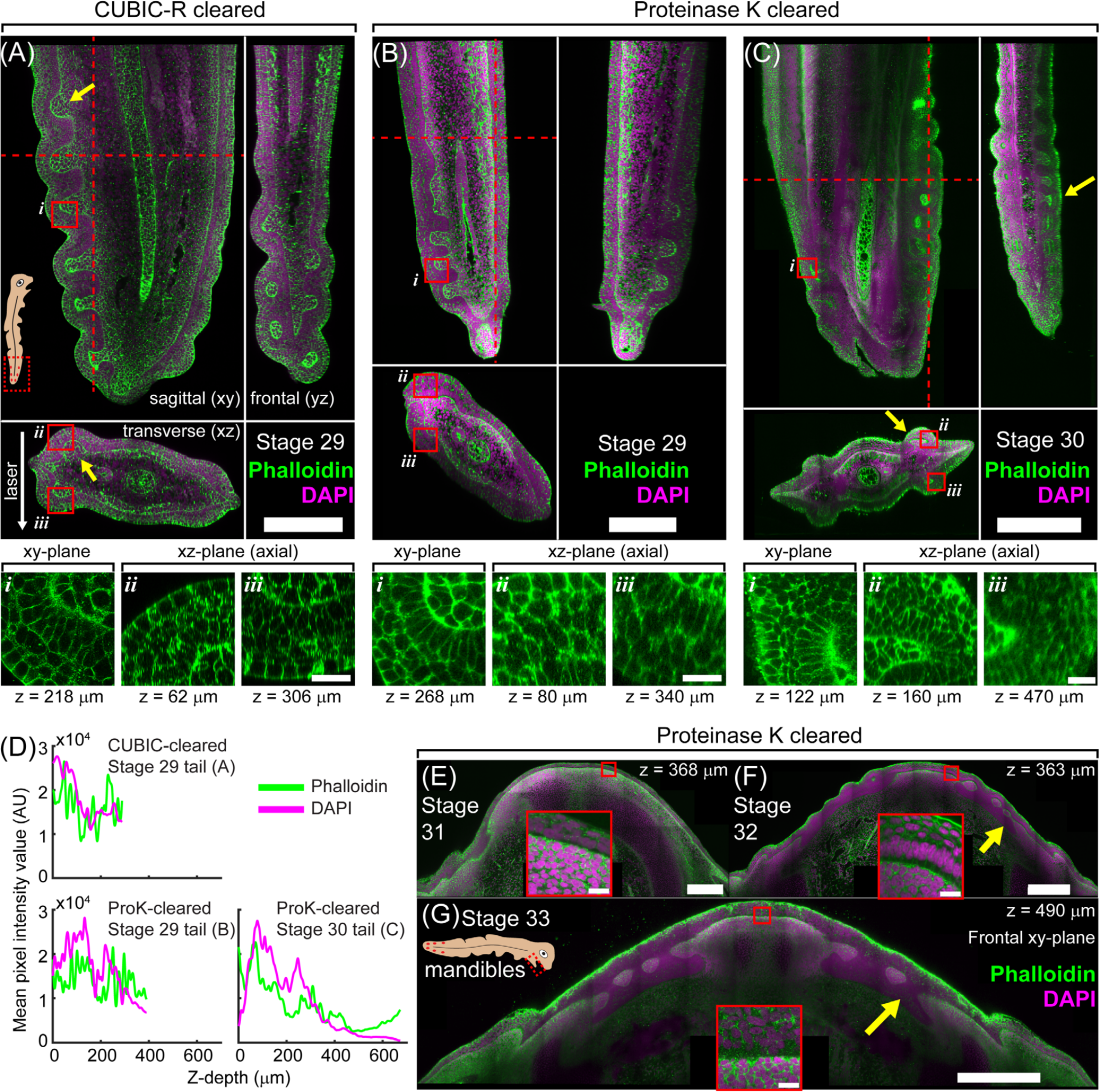
Whole tissue imaging of embryonic catsharks using MORPHOVIEW. (A–C) Orthogonal views of 3D confocal images of (A,B) stage 29 and (C) stage 30 catshark tail tips showing the dermal denticles (yellow arrows). Red lines indicate the location of the orthogonal sections. Phalloidin retention protocols were applied and tissues were counterstained with DAPI. Tissues were cleared using (A) CUBIC‐R or (B,C) Proteinase K. Zoomed‐in images show example (*i*) XY‐planes detailing the lateral resolution; and YZ‐planes indicating the axial resolution at both (*ii*) shallow and; (*iii*) deep imaging depths. The image brightness of the zoom‐in images was adjusted to showcase details of the image resolution. (D) Image intensity profile plots of the phalloidin and DAPI signals from the confocal volumes (A–C) as a function of laser penetration depth (Z‐direction). Comparison is made between the CUBIC‐R cleared stage 29 tail tissue (sample thickness: 288 μm), Proteinase K cleared stage 29 tail tissue (sample thickness: 390 μm), and Proteinase K cleared stage 30 tail tissue (sample thickness: 672 μm). (E–G) Example 3D confocal images of catshark mandibles cleared using Proteinase K. Virtual frontal slices through the mandibles showing the dental lamina (yellow arrows): (E) stage 31, (F) stage 32, and (G) stage 33 shark mandibles. All images were acquired using a 40×/1.0 water dipping objective except (A), which used a 25×/0.8 glycerol immersion objective due to the use of CUBIC‐R. Scalebars: (A,B) 300 μm; (C) 600 μm; (E) 300 μm; (F) 400 μm; (G) 500 μm; all zoomed‐in regions: 30 μm.

Protein‐gel conjugation was achieved using a heterobifunctional linker that covalently substitutes primary amines of antibodies and tissue proteins with reactive acrylamide groups, which were subsequently polymerized into the backbone of the polyacrylamide gel matrix. Using a water immersion objective, we successfully imaged catshark tail‐tip tissues, which are several hundred microns thick, at high resolution and resolving power while preserving the phalloidin signal (Figure 7B,C and Video 2). The image quality from the gel‐embedded, Proteinase K‐digested samples (Figure 7B) was comparable to that of similarly sized samples cleared with CUBIC‐R (Figure 7A), but enabled significantly greater imaging depths (Figure 7C). Moreover, the Proteinase K‐digested samples could be imaged using higher‐power objectives, significantly improving the image resolution, particularly along the axial (z) direction (Figure 7B (*iii*)).

**VIDEO 2:** 3D confocal volume of optically cleared 27 mm stage 29 catshark tail tip with phalloidin retention protocols applied, counterstained with DAPI. Animation scrolls through the sagittal slices of the 3D volume.

To compare the performance of CUBIC‐R and Proteinase K digestion tissue clearing protocols, the signal intensities of the confocal volumes were plotted as a function of laser penetration depth (Figure 7D). Signal attenuation in stage 29 shark tail tissues was similar between the two clearing protocols, with most signal intensity variations attributable to variations in tissue structure. In the larger stage 30 shark tails cleared with Proteinase K, signal attenuation became evident around 300 μm; yet the phalloidin labeling remained well resolved at deep penetration depths (Figure 7C (*iii*)).

**FIGURE 8:**
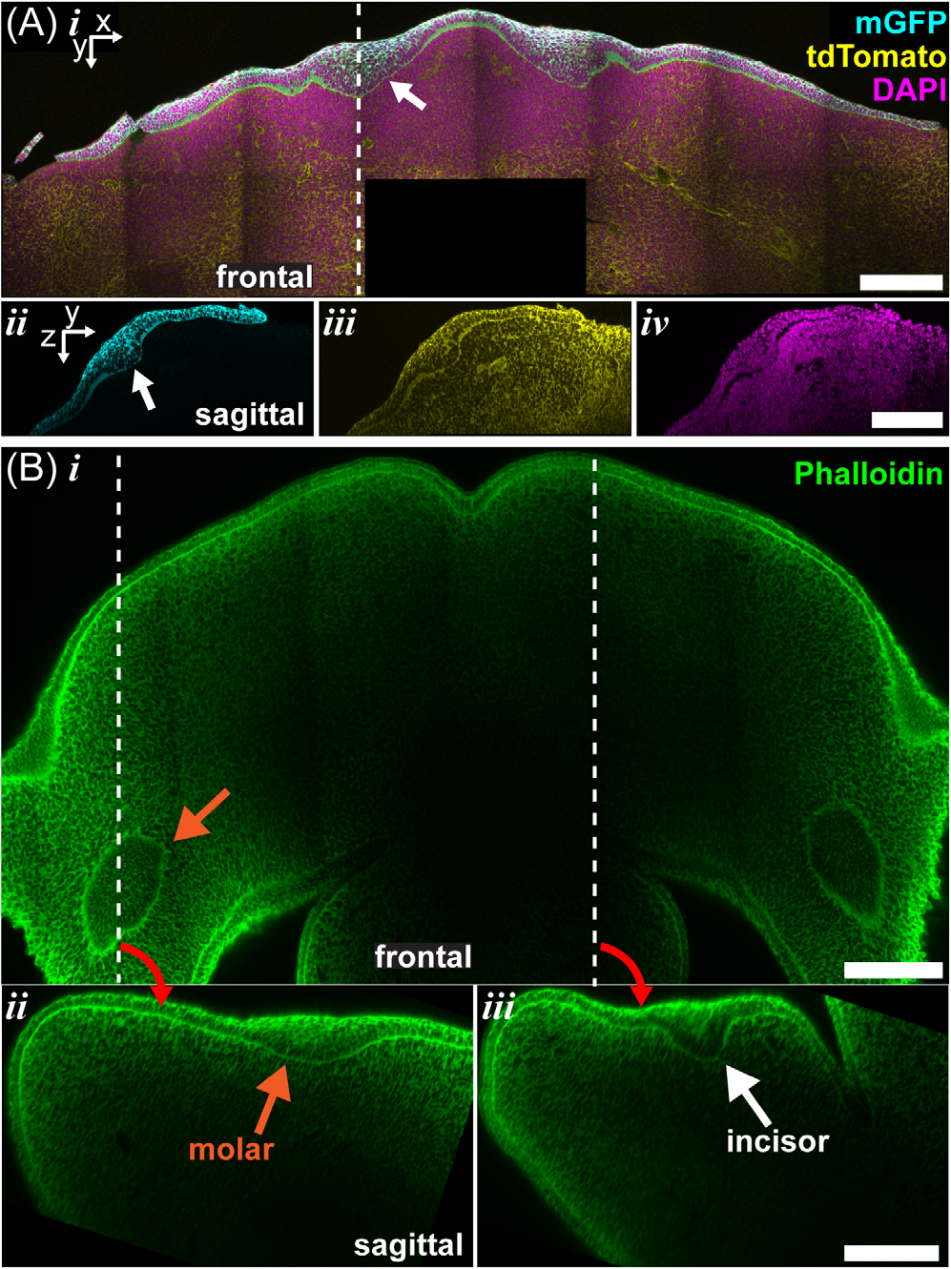
MORPHOVIEW applied to mouse mandibles. (A) Example 3D confocal volume of a E12.5 *K14* ^*Cre*^;*R26* ^*mT/mG*^ mouse mandible showing the incisor bud (white arrow) counterstained with DAPI followed by gel‐embedding and proteinase K digestion. Virtual (A*i*) frontal and (A*ii*–*iv*) sagittal slices through the confocal volume. (A*ii*) Epithelial cells are labeled by membrane GFP (mGFP), (A*iii*) mesenchymal cells retain expression of membrane tdTomato, while all cells are labeled by (A*iv*) DAPI. (B) Example 3D confocal volume of a E12.5 wild‐type mouse mandible demonstrating phalloidin retention after proteinase K digestion. Incisor buds are indicated by white arrows, and molar buds by orange arrows. Virtual (B*i*) frontal and sagittal slices through the (B*ii*) molar and (B*iii*) incisor. Dashed white lines indicate locations of sagittal slices. Gamma adjustments applied to show details. Scalebars: 200 μm.

To further evaluate the versatility of the MORPHOVIEW technique, we applied it to a range of tissues at different developmental stages. Imaging of catshark mandibles revealed detailed views of the dental lamina and developing tooth buds (Figure 7E–G). We also applied this technique to E12.5 *K14* ^*Cre*^;*R26* ^*mT/mG*^ mouse mandibles, expressing fluorescent membrane markers (Figure 8A), and to E12.5 wild‐type mouse mandibles stained with phalloidin (Figure 8B). In all instances, we were able to obtain detailed 3D images of the cell boundaries.

### 2.4 Imaging of optically cleared tissues with intact cell boundary markers enables full tissue cell segmentation and analysis of 3D cellular morphology properties

**FIGURE 9:**
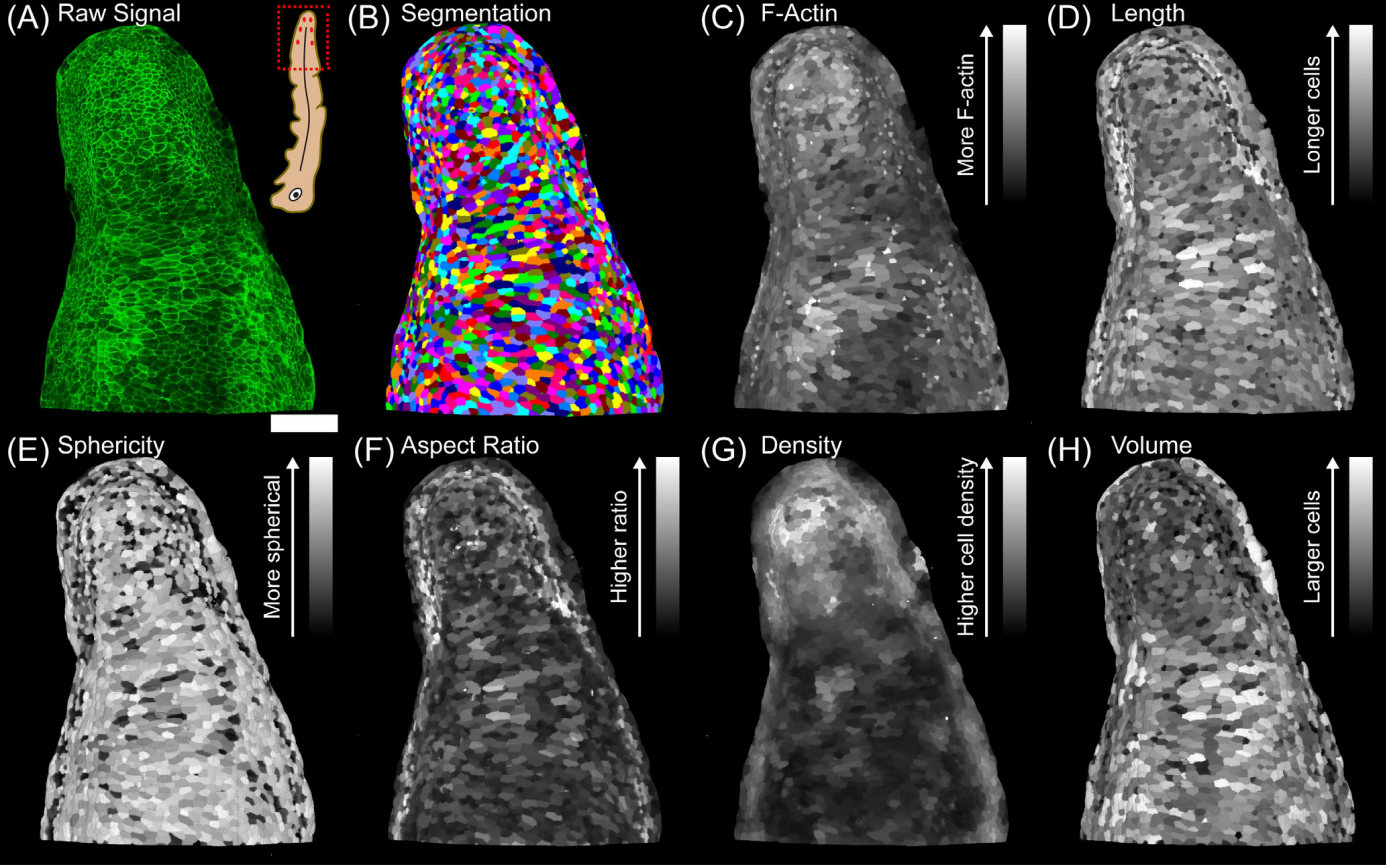
Example tail tissue from a stage 27 shark cleared and imaged to obtain 3D volumetric images and individual cell morphology properties. Volumetric images of (A) input phalloidin fluorescence signal, and (B) individually segmented cells. Heat maps of: (C) average phalloidin intensity per cell; (D) length of longest principal axis of each cell; (E) cell sphericity; (F) the aspect ratio of the largest/smallest principal axes; (G) local cell density in units of cells/volume; and (H) cell volume. Scalebar 100 μm.

We next evaluated our ability to segment individual cells following whole‐tissue confocal imaging. Using the tail tip from a stage 27 catshark as an example, we automatically segmented each cell within the tissue using a custom‐trained Cellpose segmentation model.^20^ The phalloidin channel (Figure 9A), which highlights cell boundaries, provided the primary input for the model, allowing for successful segmentation of all cells (Figure 9B and Video 3). These segmentations were then used to produce 3D heat maps quantifying various individual cell properties such as average F‐actin concentration per cell (Figure 9C); the length of the longest principal axis of the cell (Figure 9D); cell sphericity (Figure 9E); the aspect ratio of the longest and shortest principal axes of the cell body (Figure 9F); the local cell density, in units cells/unit volume (Figure 4G); and individual cell volumes (Figure 9H).

**VIDEO 3:** Animated version of Figure 4. Example tail tissue from a 24 mm (stage 27) shark cleared and imaged to obtain individual 3D volumetric cell morphology properties. Volumetric images of (A) input phalloidin fluorescence signal, and (B) individually segmented cells. (C–H) Heat maps of cellular morphology and phalloidin intensity. (C) Average phalloidin intensity per cell, (D) length of longest principal axis of each cell, E) cell sphericity, F) ratio of the largest/smallest principal axes, G) local cell density in units of cells/volume, and H) cell volume.

### 2.5 Heat maps of 3D cellular properties provide insight into epithelial bending modes

**FIGURE 10:**
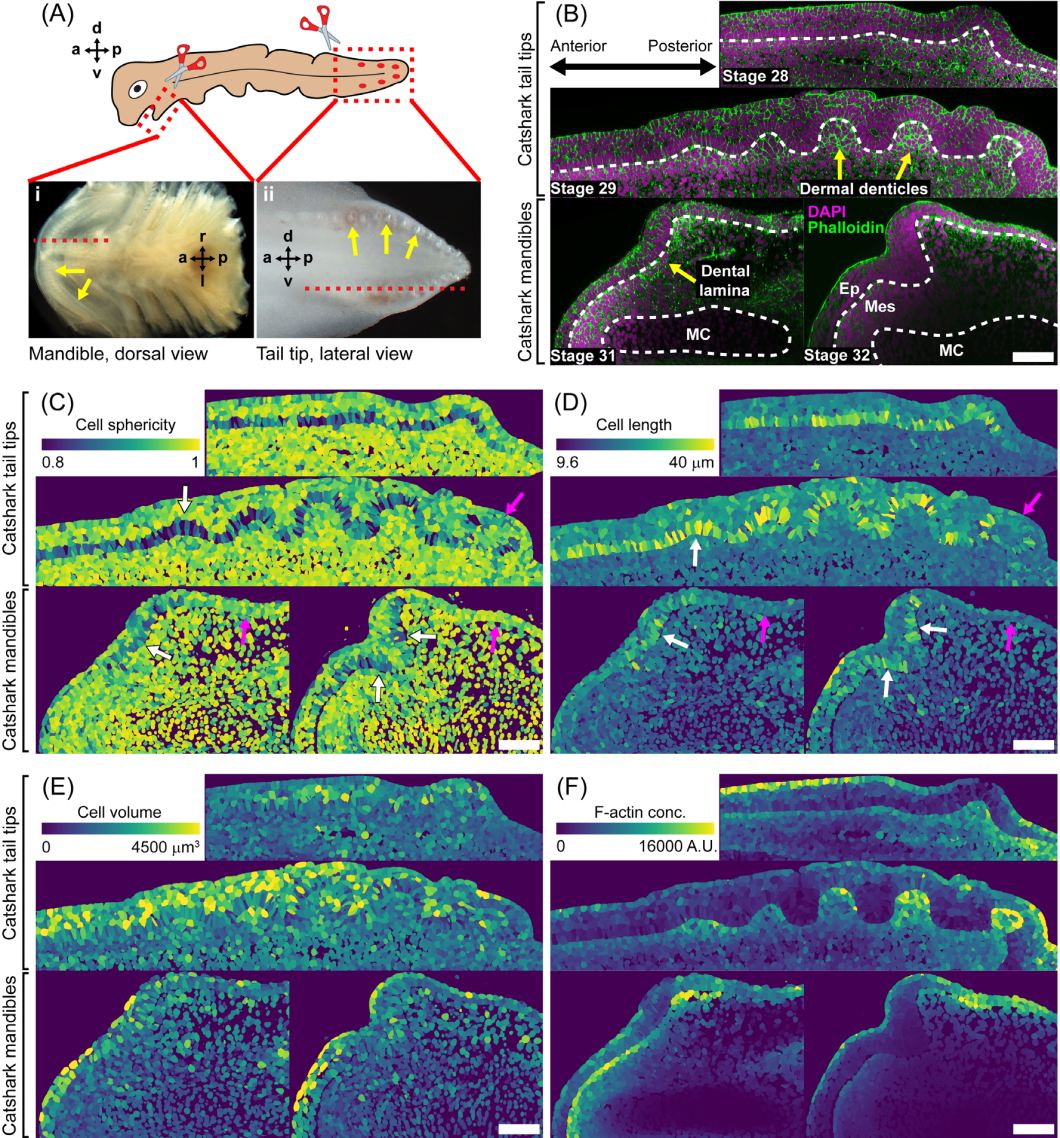
Heat maps of 3D cellular properties mapped on a cell‐by‐cell basis from 3D volumetric images. (A) Diagram of catshark (*i*) mandible showing the odontogenic band (yellow arrows) and (*ii*) tail tip showing the dermal denticles (yellow arrows). Dorsal (d), ventral (v), anterior (a), posterior (p), left (l), and right (r) anatomical directions. Red lines indicate the approximate location of the virtual sections displayed in panels (B–F). (B) Representative sections of stage 28 shark tail, stage 29 shark tail, stage 31 shark mandible, and stage 32 shark mandible stained with phalloidin and DAPI. Yellow arrows indicate the dermal denticles and dental lamina of the tail and mandible, respectively. White lines indicate the boundary between the epithelium (Ep) and mesenchyme (Mes), and the boundary of Meckel's cartilage (MC). (C–F) Cell‐by‐cell measurements of: (C) cell sphericity (unitless); (D) length of the longest principal axis of the cells; (E) cell volume; and (F) average cellular F‐actin staining intensity. White arrows indicate regions with longer and more spherical basal epithelial cells and magenta arrows indicate shorter and more elliptical basal epithelial cells. All scalebars 100 μm.

Epithelial morphogenic processes such as bending of epithelial sheets, in simple terms, can occur through two different modes: invagination into the underlying tissue, or evagination outwards from the epithelial surface.^21^ The mechanisms by which epithelial bending direction is determined, however, are not well understood. Using the catshark mandibular dental lamina and tail‐tip dermal denticles as proof‐of‐concept examples, we compared the 3D cellular properties of both invaginating (dental lamina) and evaginating (dermal denticles) tissue structures (Figure 10A). To analyze 3D cellular morphology in specific regions and orientations within the tissue interior, we generated virtual sections (Figure 10B) through the 3D cell property heat maps, providing 2D snapshots of the 3D cell properties (Figure 10C–F). To explore the cell behaviors characteristic of these two different types of epithelial bending, we examined the cell sphericity (Figure 10C), the length of the longest principal axis of the cells (Figure 10D), the cellular volume (Figure 10E), and the distribution of F‐actin across the tissues (Figure 10F).

**FIGURE 11:**
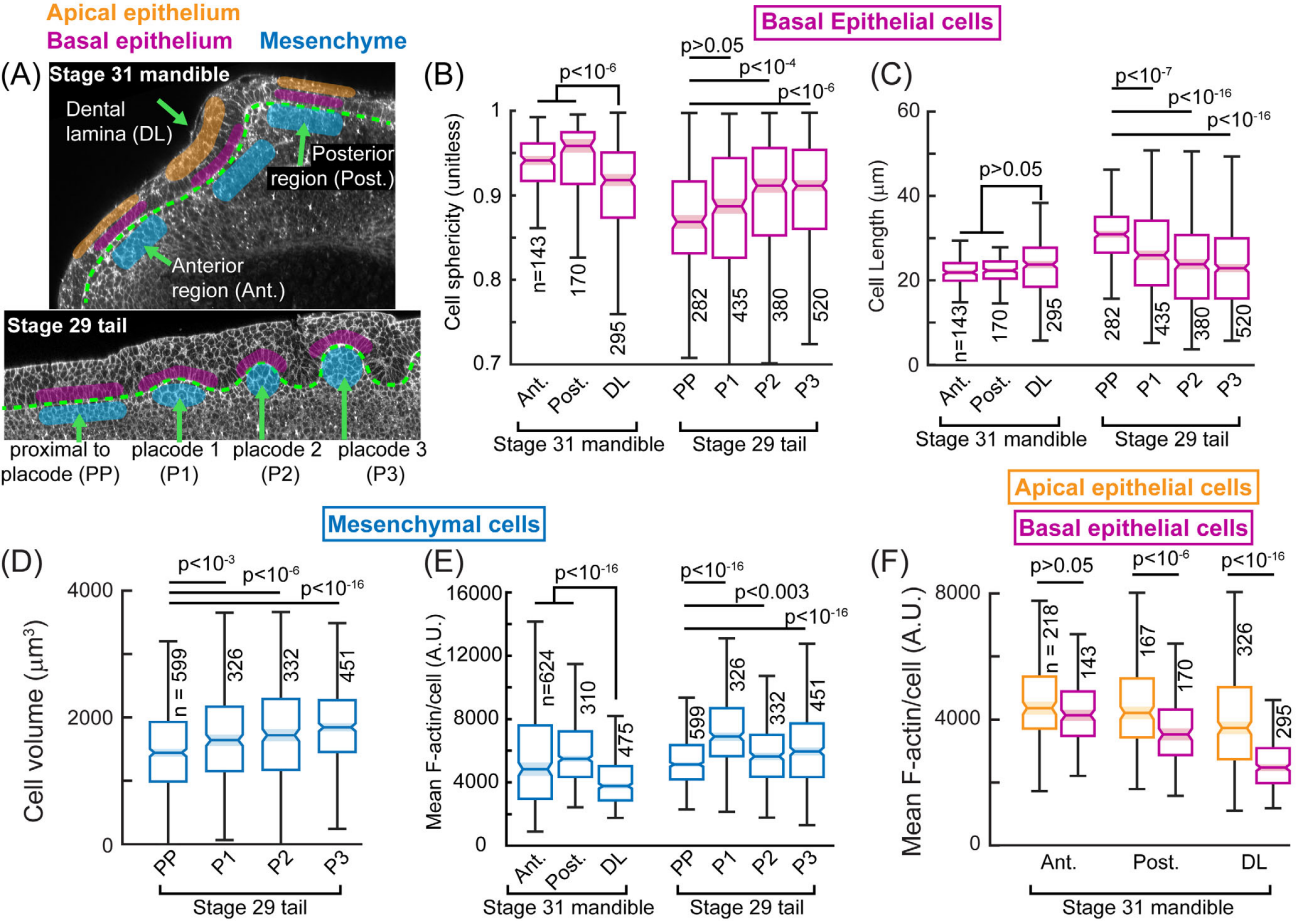
Quantification of 3D cellular properties from Figure 10. (A) Cells in the different regions of the mandible and tail tip were identified and isolated in the basal or apical epithelium and mesenchyme. The dental lamina region is compared to regions both anterior and posterior, and the three most anterior dermal denticle placodes are compared to a region proximal to the placodes. Box plots of (B) cell sphericity and (C) cell length of the basal epithelial cells. Box plots of (D) mesenchymal cell volume and (E) average cellular F‐actin staining intensity. (F) Box plots comparing the F‐actin concentration between apical and basal epithelial cells. In the boxplots, the whiskers indicate the region of nonoutliers, the edges of each box are the upper and lower quartiles, the shaded notches indicate the 5% significance level, and the line inside of each box is the sample median. Number of cells analyzed per region indicated on box plots.

While early dental lamina development is known to begin with epithelial thickening^22^ and basal epithelial cell elongation,^23^ it is less clear whether similar cell elongation precedes epithelial evagination in dermal denticles. We first confirmed that basal epithelial cells in the catshark mandibular dental lamina are significantly less spherical (Figure 10C, white arrows) than those located more anteriorly and posteriorly (magenta arrows) in both early (stage 31 shark) and later (stage 32 shark) stages of dental lamina development (Figure 11B). These less spherical cells also appear longer along their longest major principal axis (Figure 10D). Upon quantification, however, we find that in stage 31 mandibles, the basal epithelial cells in the dental lamina are not significantly longer than those more anterior or posterior (Figure 11C). In the evaginating tail‐tip dermal denticles, basal epithelial cells exhibit localized reductions in sphericity (Figure 10C) and become longer (Figure 10D) in regions of placode formation in both early (stage 28 shark) and later (stage 29 shark) stages of development. Interestingly, newly formed dermal denticle placodes (more anterior) contain basal epithelial cells that are longer and less spherical (white arrows) compared to older, more posterior placodes (magenta arrows) that have undergone greater evagination (Figure 11B,C).

To investigate whether local increases in cell volume and growth correlate with epithelial bending, we assessed cell volume in both dental lamina and dermal denticle tissues. We hypothesized that enlarged cells may contribute to convex tissue morphologies, such as in the mesenchyme of the dermal denticles or the epithelium of the dental lamina. Although no obvious spatial patterns in cell volume were observed visually (Figure 10E), statistical analysis of stage 29 dermal denticles revealed a significant trend toward increased mesenchymal cell volume within the more posterior placodes (Figure 11D).

Next, we quantified F‐actin intensity on a per‐cell basis by averaging the staining intensity across each cell's volume (Figure 10F). Mesenchymal cells underlying the dermal denticles exhibited elevated F‐actin intensity (Figure 11E), suggesting increased cell tension. By contrast, mesenchymal cells underlying the dental lamina exhibited a significantly lower F‐actin concentration than mesenchymal cells more anterior and posterior to the dental lamina (Figure 11E). Cells within Meckel's cartilage stained minimally for F‐actin (Figure 5), which, along with potential optical attenuation, contributed to the lower signal in those regions.

Previous reports have indicated that F‐actin accumulation at the apical side of cells of intestinal crypts leads to bending through apical constriction.^24^ While we did not directly measure apical vs. basal F‐actin accumulation within individual cells, we compared the accumulation of F‐actin in more apical vs. basal epithelial cells. In the dental lamina, apical epithelial cells showed higher F‐actin intensity than basal epithelial cells; however, this trend was also true in the posterior region adjacent to the dental lamina (Figure 11F), indicating that increased apical cell F‐actin is not unique to the dental lamina and thus unlikely to be the driver for invagination.

Together, these findings demonstrate that the MORPHOVIEW protocol enables quantitative analysis of cell morphology and cytoskeletal features, and supports a model in which changes in cell shape underlie both invaginating and evaginating epithelial morphogenesis.

### 2.6 Broader applications of MORPHOVIEW

One of the key advantages of the MORPHOVIEW technique is its versatility and the breadth of information that can be extracted from the resulting data sets. For example, while we primarily analyzed the mean F‐actin concentration across entire cells, the same data can be used to examine subcellular F‐actin distributions – such as apical vs. basal‐lateral enrichment, or cortical vs. medial localization within individual cells. As F‐actin is a good indicator of cellular tension, these data can further be integrated into force inference models, which estimate intercellular tensions and pressures directly from segmented cell morphologies.^25^, ^26^, ^27^ The segmented cell images generated via MORPHOVIEW are readily compatible with such models.

**FIGURE 12:**
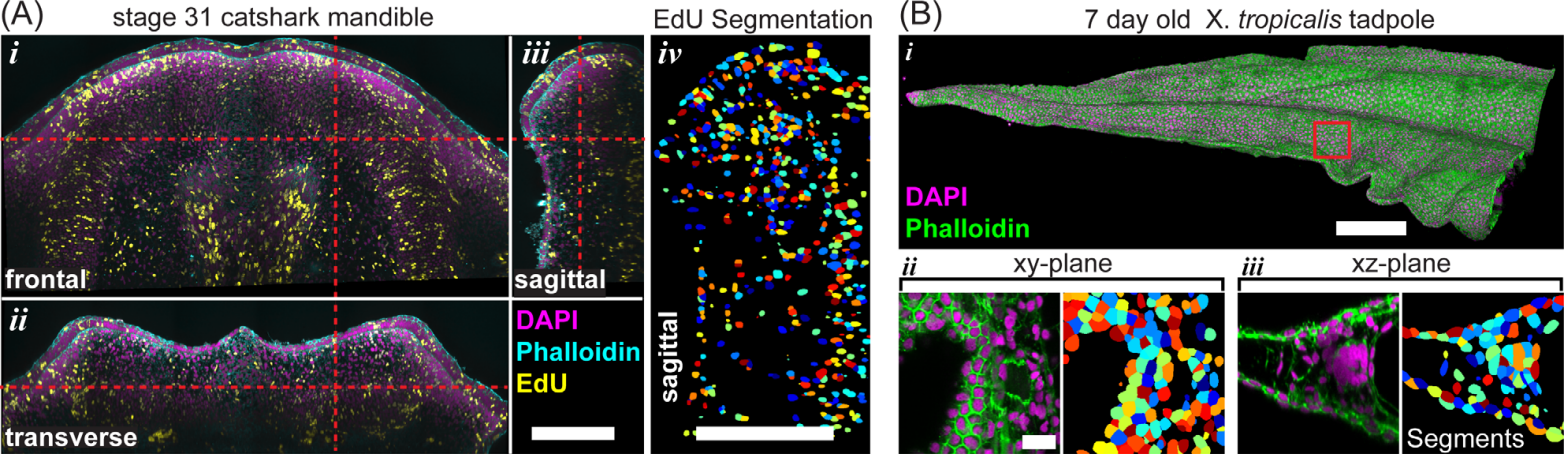
Broader applications of the MORPHOVIEW technique. (A) Stage 31 catshark incubated in EdU and then labeled using the MORPHOVIEW protocol. The mandible was dissected and a 3D image volume acquired showing the (*i*) frontal, (*ii*) transverse, and (*iii*) sagittal planes of the tissue. (*iv*) The EdU‐labeled cells were subsequently segmented using a custom‐trained Cellpose segmentation model. (B) (*i*) Tail tip from a 7 day old X. *tropicalis* tadpole was processed using the MORPHOVIEW protocol and imaged. The images were segmented in 3D using Cellpose, where both the (*ii*) xy‐ and (*iii*) xz‐planes show clear and accurate cell segmentations. All images were acquired using a confocal microscope equipped with a 40×/1.0 water dipping objective. Scalebars: (A,B) (*i*) 300 μm; (B) (*ii, iii*) 30 μm.

Beyond cell shape, the MORPHOVIEW protocol also enables analysis of nuclear shape. Nuclear morphology can be analyzed by segmenting nuclei from the DAPI channel and, for example, correlated with cellular elongation of sphericity. Additionally, since spatiotemporal patterns of proliferation play a central role in morphogenesis, it is critical to visualize where and when cells divide in 3D space. To address this, we incubated catshark embryos in 5‐ethynyl‐2′‐deoxyuridine (EdU), a thymidine analog that incorporates into the DNA during cell division and fluorescently labeled post‐fixation. Using the MORPHOVIEW protocol, we generated 3D maps of proliferating cells and segmented EdU‐positive nuclei (Figure 12A). Importantly, while EdU labeling requires copper to catalyze the click reaction – which typically disrupts phalloidin staining – we observed strong retention of phalloidin signal following EdU labeling, highlighting the robustness of the MORPHOVIEW protocol.

Since phalloidin binds F‐actin with broad cross‐species reactivity,^19^ MORPHOVIEW is potentially applicable to a wide range of model and non‐model organisms. As a phylogenetically divergent comparison to mouse and catshark, we applied MORPHOVIEW to western clawed frog (*Xenopus tropicalis*) tadpoles (Figure 12B). Without any modifications to the MORPHOVIEW protocol, we successfully stained and imaged the tail tips of 7‐day‐old tadpoles, achieving strong phalloidin signal and clear cell boundary labeling (Figure 12B (*i*)). These images were segmented using Cellpose with the same trained model used for catshark embryos (Figure 12B (*ii*, *iii*)), yielding high‐quality 3D cell segmentation in both the xy and xz (axial) planes.

## 3 CONCLUSIONS

In this work we developed a method, named MORPHOVIEW, to image cell boundaries at sub‐micron resolutions of entire tissues. While genetic model organisms can be bred to express fluorescent proteins in their cell membranes, researchers using wild type and non‐traditional model animals do not have this luxury. Given that phalloidin, which selectively labels F‐actin in most species, acts as a good marker of cell boundaries, we adapted a method to retain phalloidin staining during tissue clearing. These phalloidin‐retained tissues were further processed and optically cleared by two different methods: the embryonic tissues could simply be incubated in a RI‐matching solution, or embedded into a polyacrylamide gel, covalently binding the tissue proteins to the gel backbone, and digesting the remaining tissue so that tissue was RI‐matched with water. The latter method allowed us to utilize high magnification, long WD water dipping objectives to image the entire tissue. The subsequent images were then automatically segmented using a user‐trained neural network segmentation model, which output each individual cell volume of the tissues. We expect that the techniques devised in this work will help developmental biologists apply more quantitative measures of 3D cell morphology to understand tissue morphogenesis.

Using MORPHOVIEW, we examined epithelial bending of the invaginating catshark dental lamina and their evaginating dermal denticles. These data suggest that basal epithelial cells elongate in regions of high in‐plane epithelial compression, and that increased F‐actin activity in the mesenchymal portion of the dermal denticle placode constricts the mesenchyme, causing it to buckle outwards toward the epithelium, driving its evagination. Beyond dental biology, this technique offers broad applicability to studying tissue development and morphogenesis across various systems. Biologists quantifying cellular properties such as morphology, position, number, RNA or protein expression require accurate segmentation of cell borders in 3D space. The method described here enables researchers to analyze individual cells within entire tissues from a wide range of both model and non‐model organisms.

## 4 EXPERIMENTAL PROCEDURES

### 4.1 Materials

Tricaine (Syndel), Phosphate Buffered Saline (PBS) (Fisher Scientific), paraformaldehyde (PFA, Thermo Scientific), goat serum (Gibco), bovine serum albumin (BSA, Fisher Scientific), Triton X‐100 (Fisher Scientific), phalloidin conjugated to either Alexa Fluor™ 488 (Thermo Fisher A12379) or Alexa Fluor™ 633 (Thermo Fisher A22284), 4′,6‐diamidino‐2‐phenylindole (DAPI, Biotium 40009), rabbit anti‐Alexa Fluor™ 488 (Thermo Fisher A11094), 35 mm glass bottom dish (Thermo Scientific), low melting point agarose (Thermo Scientific), #1.5 coverglass (Fisher Scientific), dimethyl sulfoxide (DMSO, Thermo Scientific), acrylamide (AAm, Thermo Scientific), bis‐acrylamide (BAAm, Thermo Scientific), ammonium persulfate (APS, Acros Organics), tetramethylethylenediamine (TEMED, Thermo Scientific), 4‐hydroxy‐2,2,6,6‐tetramethylpiperidin‐1‐oxyl (TEMPO, Thermo Scientific), and Proteinase K (Invitrogen) were used as purchased.

A wash solution of 0.1% Triton X in 1× PBS (PBST), CUBIC‐R RI‐matching solution (45 w/v% antipyrine [Thermo Scientific], 30 w/v% nicotinamide [Thermo Scientific], and 0.5 v/v% N‐butyldiethanolamine [Thermo Scientific]) in distilled water as described previously^18^ was made fresh in the lab. Digestion buffer consisting of Triton X, ethylenediaminetetraacetic acid (EDTA, Sigma), tris(hydroxymethyl)aminomethane (Tris hydrochloride, Roche), and sodium chloride (Sigma) was prepared as previously described.^17^

6‐((Acryloyl)amino)hexanoic Acid, Succinimidyl Ester (AcX, CAS number: 63392‐86‐9) was synthesized as previously described.^28^, ^29^

### 4.2 Collection of embryos and fixation

#### 4.2.1 Mice

*K14* ^*Cre*^;*R26* ^*mT/mG*^ mice were generated by crossing *K14* ^*Cre*^ (MGI:2445832)^30^ with *R26* ^*mT/mG*^ (MGI:3716464).^31^ C57BL/6 were purchased from JAX. All mice were group housed and genotyped as previously published. To generate embryos for experiments, mice were mated overnight, and noon of the day of vaginal plug discovery was designated as E0.5. Pregnant females were euthanized by CO_2_ followed by cervical dislocation. Both male and female GFP‐positive *K14* ^*Cre*^;*R26* ^*mT/mG*^ embryos or C57BL/6 embryos were selected at random and used for experiments. Mandibles were dissected out in PBS and fixed in freshly made 4% paraformaldehyde (PFA) in 1× PBS for 12 h on a rocker at 4°C. The samples were then washed in 1× PBS three times for 10 min each and stored in 0.5% PFA at 4°C until use. All mice were maintained in the University of California Los Angeles (UCLA) pathogen‐free animal facility. All animal procedures were conducted in compliance with animal protocols approved by the UCLA Institutional Animal Care and Use Committee (Protocol Number ARC‐2019‐013).

#### 4.2.2 Sharks

Fertilized eggs of small spotted catshark (*Scyliorhinus rotifer*) were obtained from the Marine Biological Laboratory, Woods Hole, MA (stages 28–32). Embryos were mechanically extracted and placed in a 0.5% Tricaine solution diluted in 1× PBS solution for 10 min. Embryos were then fixed in freshly made 4% PFA in 1× PBS for 24 h on a rocker at 4°C. Fixed samples were washed in 1× PBS four times for 1 h each. Mandibles and tails were dissected out and stored in 1× PBS. From this point forward, samples were protected from light. All animal procedures were conducted in compliance with animal protocols approved by the University of Chicago Institutional Animal Care and Use Committee (IACUC# 71033‐05).

#### 4.2.3 Frogs

*X. tropicalis* (NXR_1018) NF57 tadpoles were purchased from the National Xenopus Resource (NXR, Marine Biological Laboratory RRID:SCR_013731). Upon receiving, tadpoles were placed in a 0.5% Tricaine solution diluted in 1× PBS solution for 10 min. Tadpoles were then fixed in freshly made 4% PFA in 1× PBS for 24 h on a rocker at 4°C. Fixed samples were washed in 1× PBS four times for 1 h each. Tails were dissected out and stored in 1× PBS. From this point forward, samples were protected from light. All animals were maintained and used in accordance with protocols approved by the University of Chicago Institutional Animal Care and Use Committee (IACUC; permission no. 72696).

### 4.3 Immunofluorescence labeling

#### 4.3.1 Thin sectioning

Fixed tissues were stepped into 30% sucrose before embedding in OCT and cryosectioning (Thermo Fisher NX50) 10 μm thick sections. Sections were dried and stored at −80°C. Sections were rehydrated in PBS before use.

- Antibody and fluorescence labeling. Sections were incubated in blocking buffer (5% goat serum, 1% BSA, 0.3% Triton X in 1× PBS) for 1 h. The sections were then incubated in primary antibodies overnight at 4°C: monoclonal rabbit anti‐PMCA1 (1:200, abcam, ab190355); monoclonal mouse anti‐alpha 1 sodium potassium ATPase antibody (1:200, abcam, ab7671); monoclonal rabbit anti‐pan cadherin antibody, intercellular junction marker (1:200, abcam, ab51034); and monoclonal rabbit anti‐alpha 1sodium potassium ATPase antibody (1:200, abacm, ab76020) in blocking buffer. Sections labeled with anti‐pan actin (1:300, Cytoskeleton, AAN02) were labeled according to the manufacturer's protocol. The next day, the sections were washed with PBS and incubated in secondary antibodies (1:300, goat anti‐rabbit 633, Biotium 20,122) and DAPI (1:500) in blocking buffer for 2 h at room temperature. Sections were then washed with PBS and mounted with coverglass.
- Membrane labeling. To test different strategies to label cell membranes, thin sections were labeled with either a DiI‐based lipophilic dye (CellBrite Orange, Biotium 30,022) or fluorescent WGA (WGA CF555, Biotium 29,076). Sections stained with CellBrite were permeabilized with 0.1% Triton‐X in PBS for 10 min (if noted), washed with PBS, incubated in CellBrite (1:300 in PBS) for 10 min, washed with PBS, and mounted in CUBIC‐R or PBS. Sections stained with WGA were incubated in WGA (1:200 in PBS) for 30 min, washed with PBS, post‐fixed in 1% PFA in PBS for 20 min, permeabilized with 0.1% Triton‐X in PBS for 30 min, washed with PBS, and mounted in PBS or CUBIC‐R.
- Anti‐Alexa Fluor™ 488 labeling. To test the ability to retain phalloidin labeling using thin sections, sections were incubated in blocking buffer (5% goat serum, 1% BSA, 0.3% Triton X in 1× PBS) for 1 h, followed by phalloidin‐Alexa Fluor™ 488 (1:200, ThermoFisher, A11094) in blocking buffer overnight at 4°C. Sections were washed with PBST. At this point, positive controls were mounted in PBS, and negative controls were mounted in CUBIC‐R with no further primary or secondary antibodies. Sections were then incubated in anti‐fluorophore antibody (1:200, ThermoFisher, A11094) in blocking buffer overnight at 4°C. Samples without secondary antibody labeling were mounted in PBS or CUBIC‐R at this point. Sections were then incubated in secondary antibodies (1:300) in blocking buffer for 2 h. Sections were mounted in CUBIC‐R.

#### 4.3.2 Whole mount antibody and fluorescence labeling

Mouse mandibles, shark mandibles and tails, and tadpole tails were first incubated in blocking buffer (5% goat serum, 1% BSA, 0.3% Triton X in 1× PBS) for 24 h on a rocker at 4°C. Next, the samples were incubated in phalloidin conjugated to either Alexa Fluor™ 488 or 633 (1:200) in blocking buffer for 7 days on a rocker at 4°C. The samples were then washed in PBST three times for 1 h each before storage in PBS. Samples receiving no further antibody treatment were either cleared or imaged directly. Samples undergoing the MORPHOVIEW protocol were stained with anti‐fluorophore antibody (1:200, ThermoFisher, A11094) in blocking buffer for 3 days on a rocker at 37°C protected from light. The samples were then washed in PBST three times for 1 h each. The labeled samples were then stained with secondary antibody (1:300, goat anti‐rabbit 633, Biotium 20122) in blocking buffer for 3 days on a rocker at 37°C. The samples were then washed in PBST three times for 1 h each.

#### 4.3.3 Whole mount 5‐ethynyl‐2′‐deoxyuridine (EdU) staining

EdU labeling was performed according to the manufacturer's protocol (Click‐iT™ EdU Cell Proliferation Kit, Alexa Fluor™ 488 dye, ThermoFisher C10337). Briefly, live shark catshark embryos were mechanically extracted from their egg case and carefully placed in a six‐well plate containing 8 mL of 0.5 mg/mL EdU solution diluted in salt water. The shark embryos were incubated in the EdU solution for 7 days, and 4 mL of the EdU solution was replaced every 2 days. The embryos were sacrificed in a 0.5% Tricaine solution diluted in 1× PBS solution for 10 min. Embryos were then fixed in freshly made 4% PFA in 1× PBS for 24 h on a rocker at 4°C. Fixed samples were washed in 1× PBS four times for 1 h each. Mandibles and tails were dissected out and stored in 1× PBS. The tissues then went through the whole mount antibody and fluorescence labeling procedure described above. After the conclusion of the secondary antibody labeling, the tissues were incubated in 1 mL of the Click‐iT® reaction cocktail and incubated overnight at RT while rocking. The samples were washed with 3% BSA in PBS and cleared using the gel‐embedded, Proteinase K tissue clearing procedure outlined below.

### 4.4 Tissue clearing

Tissues were rendered optically transparent by two different methods: the first renders the samples optically transparent by simply immersing them in an aqueous‐based high RI solution; the second covalently binds the antibodies into a 3D polymer network and enzymatically digests the remaining tissue.

- To render tissues optically transparent in a high RI solution, fluorescently labeled samples were first fixed in freshly made 1% PFA in 1× PBS for 24 h on a rocker at 4°C. Samples were then washed for 2 h in 1× PBS and counterstained with DAPI (0.1 mg/mL) for 24 h on a rocker at 4°C. Samples were then washed for 2 h in 1× PBS before immersing in CUBIC‐R for 1–3 days (depending on the size of the embryonic sample) to render the tissues optically transparent for 3D imaging. Samples were mounted on a 35 mm coverglass bottom dish suspended in a 0.5% agarose solution in CUBIC‐R to avoid movement of the sample during the imaging process. The mounted samples were then covered with a coverglass before imaging.
- To render tissues optically transparent in a low RI aqueous solution, fluorescently labeled samples were incubated in a freshly made solution of the heterobifunctional linker AcX. A stock solution of 50 mg/mL AcX in anhydrous DMSO was freshly prepared before diluting 10‐fold in PBS. Samples were immediately immersed in the AcX solution for 24 h on a rocker at 22°C. The functionalized tissues were then washed with PBST for 2 h and immersed in a cold polyacrylamide pre‐polymer solution consisting of AAm (9.6 w/v%), BAAm (0.25 w/v%), TEMPO (0.75 w/v%), APS (0.2 w/v%), and TEMED (0.2 v/v%) in 1× PBS for 4 h in an ice bath, while rocking. The pre‐polymer solution was replaced and the samples were placed between two glass slides with 1 mm spacers, which were filled with the pre‐polymer solution. The glass slide‐sample sandwiches were then incubated at 37°C for 1 h to polymerize the polyacrylamide gels. The tissue‐gel construct was then trimmed and immersed in a solution of Proteinase K in digestion buffer (0.2 mg/mL) and DAPI (0.1 mg/mL) and incubated for 48 h on a rocker at 22°C, or until the tissue was rendered optically transparent. The tissues were then washed with 1× PBS three times for 1 h each. Samples were mounted in a 35 mm plastic dish suspended in a 0.5% agarose solution in 1× PBS before imaging.

### 4.5 Confocal imaging

Samples were imaged with a Zeiss confocal laser scanning microscope (LSM 900) equipped with either a Plan‐Apochromat 25×/0.8 glycerine immersion objective with a WD of 0.57 mm or a Plan‐Apochromat 40×/1.0 DIC VIS‐IR water dipping objective, WD 2.5 mm. A pinhole of 1 AU was used. Three‐dimensional, whole‐tissue volumes were acquired using a combination of Z‐stacks and image tiling. Tissues cleared with CUBIC‐R were imaged using the 25× objective and gel‐embedded tissues cleared with Proteinase K were imaged using the 40× objective.

### 4.6 Image processing and segmentation

For full details on the computational methods used, see the Supplementary Information. We provide a detailed set of instructions on how to stitch large tiled 3D image stacks, cell segmentation training and implementation, and methods to analyze cell morphology and other properties from the segmented images. The MATLAB code, Cellpose model, and an updated version of the computational methods are available at https://github.com/snoreis/MORPHOVIEW. We briefly describe the procedure below.

Images were processed and visualized with Zen (Zeiss), ImageJ, MATLAB, and Imaris. Multi‐tiled images were stitched with the ImageJ plugin BigStitcher^32^ where the images were resampled to have cuboidal voxel sizes. At times, contrast adjustments were applied to enhance details for visualization and segmentation, but not for signal quantification. The stitched images were reoriented and cropped to contain the region of interest and reduce file size and were segmented using CellPose.^14^ Before segmentation, the neural network model was trained using a subset of images where the phalloidin signal was used as the primary channel, and the DAPI signal was used as the secondary.^20^ Training was repeated and refined as needed to produce properly segmented 3D cell volumes. Segmented volumes were output as multi‐Z masks tiff file.

Once the individual cells were segmented, the mask files were imported into MATLAB, where the properties of 3D volumetric image regions were quantified using the regionprops3 algorithm. We define the cell volume as the total number of voxels belonging to the segmented cell region. The F‐actin concentration is the mean of all the per‐voxel intensity values of the phalloidin channel within the segmented cell region. The segmented cell regions are then approximated as ellipsoids that have the same normalized second central moments as the segmented cell region. From these ellipsoids, the three major axes are calculated from longest to shortest. The cell length is defined as the length of the longest principal axis. The aspect ratio is the ratio of the longest and shortest principal axes. The object sphericity (Ψ) is defined as $\Psi =\pi^{1/3}{\left(6V\right)}^{2/3}/A$, where *V* is the volume and *A* is the surface area of the approximated ellipsoid. The sphericity of a sphere is 1. A piece of custom MATLAB code was then utilized to reconstruct 3D heatmaps of the segmented cells labeled with different morphological features (volume, sphericity, F‐actin concentration, cell length, etc. …). To calculate the signal intensity profile of the 3D volumes, we used the ImageJ Plot Profile tool.

### 4.7 Statistical analysis

All statistical calculations were performed in Origin(Pro), OriginLab Corporation. All plots were made in MATLAB and Adobe Illustrator. In the boxplots, the whiskers indicate the region of nonoutliers, the edges of each box are the upper and lower quartiles, the shaded notches indicate the 5% significance level, and the line inside of each box is the sample median. To determine the degree to which the population means were different, we applied a one‐way ANOVA test. For each pair of samples, the p value was calculated (Tukey method) as a measure of the difference between the two sample populations. Due to the sample size in this work, we used the *p* value of .01 as a threshold for statistical significance but examined the *p* value more generally as an indication of the degree to which the sample means were different.

## Supporting information


**Data S1.** Supporting Information.
