# Supplementary material for: Whole tissue imaging of cellular boundaries at sub‐micron resolutions for deep learning cell segmentation: Applications in the analysis of epithelial bending of ectoderm
Source: Dev Dyn. 2025 Jul 26;255(5):503–21. doi: 10.1002/dvdy.70061 (PMC12818342; doi:10.1002/dvdy.70061)
Supplement: Supplementary file 4 — Data S1. Supporting Information. [file DVDY-255-503-s001.docx]

Supporting Information

**Whole Tissue Imaging of Cellular Boundaries at Sub-Micron Resolutions for Deep Learning Cell Segmentation: Applications in the Analysis of Epithelial Bending of Ectoderm**

Sam Norris^1^, Jimmy K. Hu ^2,3, *^, Neil H Shubin^1, *^

^1^ Department of Organismal Biology and Anatomy, The University of Chicago, Chicago, IL, USA

^2^School of Dentistry, University of California Los Angeles, Los Angeles, California, USA

^3^Molecular Biology Institute, University of California Los Angeles, Los Angeles, California, USA

^*^Corresponding author(s)

**Supporting Computational Methods**

The purpose of this supplementary information is to provide a detailed guide on the data processing and computations utilized in this protocol. The MATLAB files (which can be found in the GitHub: <https://github.com/snoreis/MORPHOVIEW>) read the segmented cell data (we use [Cellpose](https://github.com/MouseLand/cellpose)) and calculate different cell shape parameters on a cell-by-cell basis. Since the purpose of this pipeline is to analyze large 3D images, a CUDA-capable NVIDIA GPU was utilized and is **highly** recommended. To utilize the GPU with python, the NVIDIA [driver](https://www.nvidia.com/en-us/drivers/) for the GPU must be installed, and we also recommend installing the CUDA toolkit.

For any questions, we recommend [posting to the GitHub page](https://github.com/snoreis/MORPHOVIEW/discussions), or contacting the authors directly.

**Image Stitching**

For large, multi-tile confocal images such as those presented in this protocol, we find that images need to be stitched using tile translations in all three X, Y, and Z directions, and sometimes rotations around these axes. The most useful program we've found thus far is the ImageJ/FIJI plugin [BigStitcher](https://imagej.net/plugins/bigstitcher/).


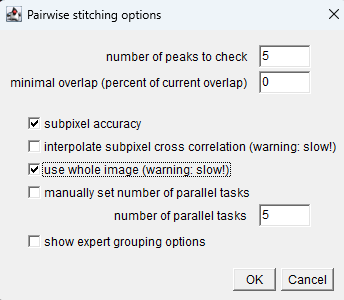
The easiest way to import files into BigStitcher is to use the [**Automatic Loader**](https://imagej.net/plugins/bigstitcher/autoloader) which is BioFormats based, and can thus read raw microscope-specific files (.czi, .lif, .nd2, etc…).

**Calculate pairwise shifts**

We found that the [phase correlation method](https://imagej.net/plugins/bigstitcher/advanced-stitching#advanced-pairwise-shift-calculation) to find the pairwise shifts to stitch the tiles works best and has a number of parameters that can be tweaked between different image sets. We used the following parameters with high success:

number of peaks to check: **5**

minimal overlap: **0**

subpixel accuracy: **Yes**

interpolate subpixel cross correlation: **No**


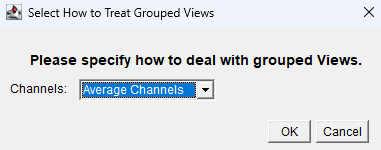
use whole image: **Yes**

In the next step, you can either choose a single channel, or average all channels to perform the stitching. This depends on the image, and should be adjusted to get the best stitching.


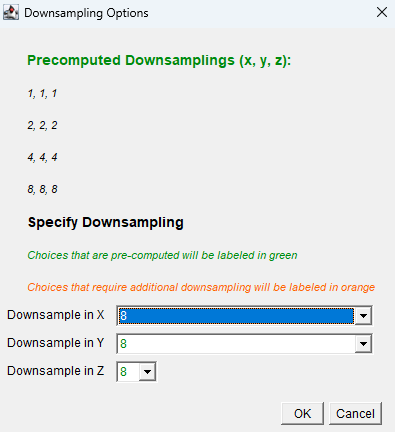


Next, BigStitcher asks if you want to down sample the image, which we highly recommend. In this protocol, we iteratively stitched the images, starting with highly down sampled images (8 in each direction) followed by a down sampling factor of 4, then 2. Whichever down sampling factor that produces the best results should be used for final image fusion.

**Apply Shift**

After the pairwise shifts have been calculated, the shift must be applied. We use the default settings:

[Optimize globally and apply shift](https://imagej.net/plugins/bigstitcher/global-optimization#simple-mode) -> Simple mode -> Two-Round: Handle unconnected tiles, remove wrong links RELAXED (5.0x/7.0px)

**Image fusion**


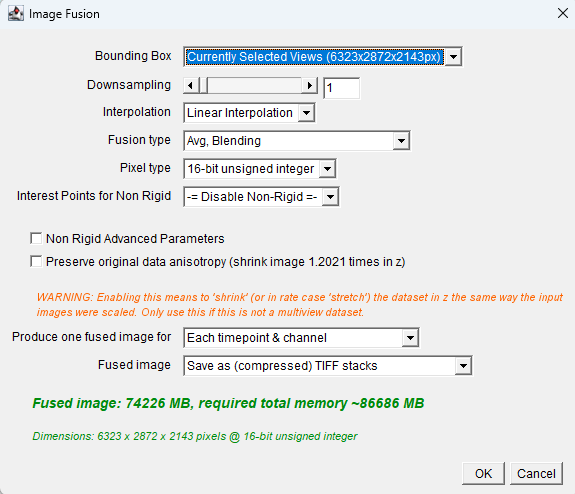
Next, the tiles must be fused together into a single image. We use the [Advanced Fusion window](https://imagej.net/plugins/bigstitcher/fuse#advanced-fusion). After selecting all the tiles that we wish to stitch, the following parameters were utilized:

Bounding box: **Currently selected views**

Downsampling: **1**

Interpolation: **Linear interpolation**

Fusion type: **Average blending**

Pixel type: **16-bit**

Interest points for non rigid: **Disable**

Non rigid advanced parameters: **No**

Preserve original data anisotropy: **No**

Produce one fused image for: **Each timepoint & channel**

Fused image: **Tiff stacks**

From there, the default options/values are used in the following prompts, which produces single channel stitched images in the Tiff format that can be combined in ImageJ. These large images can be input into Cellpose as whole images, or cropped before Cellpose segmentation.

**Cellpose Segmentation**

[Cellpose](https://github.com/MouseLand/cellpose) is a great way to segment cells from a 3D image volume as long as your cells have a nice cell boundary marker. Again, we refer you to the Cellpose github for updated installation instructions. In brief, we run the following in Anaconda:

conda create --name cellpose python=3.10

conda activate cellpose

python -m pip install cellpose[all]

pip uninstall torch

conda install pytorch pytorch-cuda=11.8 -c pytorch -c nvidia

In most cases, the Cellpose GUI can be used to analyze the images:

python -m cellpose --Zstack.

Images were directly loaded into the GUI using the default parameters:

'lowhigh': None,

'percentile': [1.0, 99.0],

'normalize': True,

'norm3D': False,

'sharpen_radius': 0,

'smooth_radius': 0,

'tile_norm_blocksize': 0,

'tile_norm_smooth3D': 1,

'invert': False


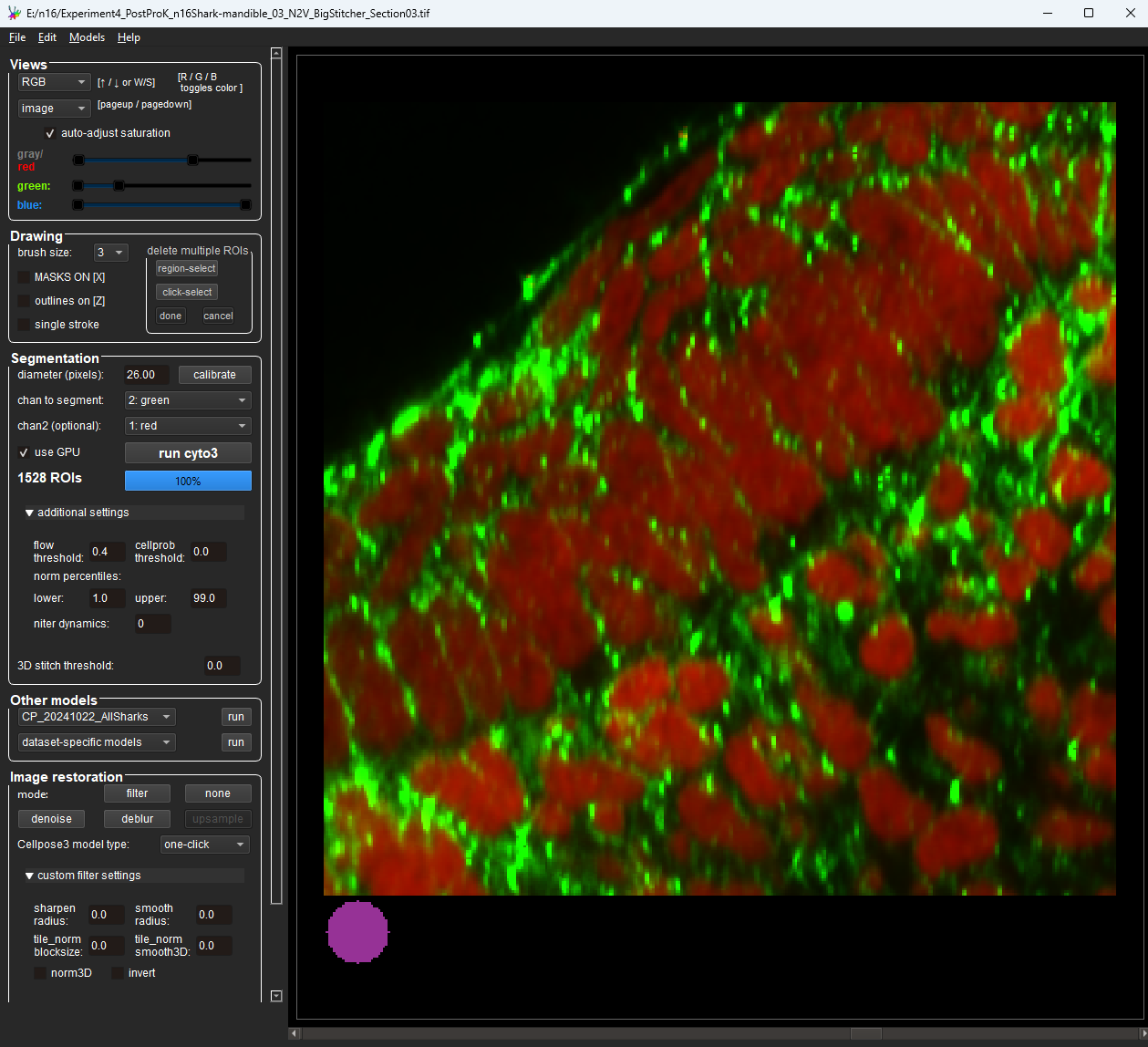


**Model Training**

In order to properly segment the images, we trained our own cellpose model. Details of how to train your own model can be found in this article: <https://www.nature.com/articles/s41592-022-01663-4>, or the [Cellpose documentation](https://cellpose.readthedocs.io/en/latest/train.html). The trained model used for the protocol described here (CP_20241022_AllSharks) can be found in the protocol GitHub: <https://github.com/snoreis/MORPHOVIEW>

For the samples analyzed in this work, we trained a single model incorporating manually segmented images from all image volumes presented in this work. Ideally, the image resolution should be high enough that a single cell is ~30 voxels in diameter, which we found to correspond to a ~0.5 μm voxel size. From each image volume, cropped, 2D “training images” from all three XY, XZ, and YZ image planes were created, segmented, and used to produce the trained model. For image volumes with Z-dimensions more than ~400 μm, we found it important that the XZ and YZ training images contain the full Z-span of the volume since some light attenuation occurs, and the Cellpose model should be trained on images with a wide range of image intensity. The process of manual image segmentation was iterative: image volumes were segmented using the custom model, analyzed for regions that produced poor segmentation results, followed by further manual segmentation of training images in these regions, and retraining of the model. In total, 144 training images were segmented, each containing ~50-200 cells. Training was performed starting with the Cellpose built-in “Cyto3” model (which helps improve the model’s generalization), a learning rate of 0.1, a weight decay of 0.0001, and 200,000 epochs. To assess the model’s performance on unseen data and to test for overfitting, the model was tested on an image volume that did not contribute to the training. Given the model’s good performance on unseen data, we did not take any additional steps to improve generalization and mitigate overfitting such as augmentation via rotation, scaling, flipping, elastic deformations, or other modifications.

**Segmentation**

In Cellpose, we used the following (default) parameters for segmentation:

Diameter: 26

Flow threshold: 0.4

Cellprob threshold: 0.0

Niter dynamics: 0

3D stitch threshold: 0.0

Perhaps the two most important factors that can be adjusted for optimal cell segmentation are diameter and 3D stitch threshold:

The diameter, which should be approximately the diameter of an average cell in pixles/voxels, is required by Cellpose to properly scale the images to match the model. Changing the diameter will change the results that the algorithm outputs. When the diameter is set smaller than the true size then cellpose may over-split cells. Similarly, if the diameter is set too big then cellpose may over-merge cells. Cellpose has an automated estimation of the diameter, which we find to work well in most cases. Images used in this work had a voxel size of 0.42 µm; thus a diameter of ~30 worked well in all images.

The 3D stitch threshold: computes masks in 2D then stitch together masks across the planes. The default 3D segmentation in the GUI is 2.5D segmentation, where the flows are computed on each YX, ZY and ZX slice and then averaged, and then the dynamics are run in 3D. Instead if 3D stitch threshold > 0.0 Cellpose will create ROIs in 2D on each XY slice and then stitch them across slices if the IoU between the mask on the current slice and the next slice is greater than or equal to the stitch_threshold. In some cases, especially if differences in YZ and XZ slices that make them unable to be used for 3D segmentation, stitching the 2D XY slices produces better results.

After segmentation, the masks/segments should be saved as a 3D PNG/TIFF.

**MATLAB Analysis**

**INSTALLATION** Use of the included .m files requires MATLAB to be installed.

The main MATLAB file from which everything is run is **MAIN.m**

**Data loading**

We first load the data in the form of a 3D TIF image file.

BaseFileName = "n42/convextest/Experiment1_PostProK_n42Shark_01_Section1_Stitched";

%Raw Image

RawImage = tiffreadVolume(BaseFileName + "_C2.tif");

%Corresponding segmented cells

A = tiffreadVolume(BaseFileName + '_masks_04.tif');

This code loads both a raw image data and the corresponding masks file. Note: in this example we load only Channel 2, which includes a signal (antibody, phalloidin, DAPI) that we want to quantify in each segment. Loading the RawImage is optional.

**Measure properties of 3D volumetric image regions**

Next, we use MATLAB's built-in [regionprops3](https://www.mathworks.com/help/images/ref/regionprops3.html) to measure a set of properties for each connected component (object) in the 3-D volumetric image. The output masks/segments from cellpose can be automatically loaded into regionprops3 without any modification.

%% Calculate region properties

B = regionprops3(A, RawImage, "ConvexHull", "Centroid", "Volume", "MeanIntensity", "PrincipalAxisLength", "ConvexVolume", "Solidity", "BoundingBox", "ConvexImage", "SurfaceArea");

Here we have included all properties necessary to run every type of cell shape analysis included. However, if you are only interested in, for example, cell volume, B = regionprops3(A,"Volume") would be enough and run much faster. regionprops3 can be quite slow for very large images.

**Create convex hull-based objects**

Although not necessary, the segmented image can be "smoothed" by converting all the cells into convex hulls, then rewriting the data. We find that the cellpose output of 3D data can be a bit on the noisy side and fragmented, especially if the input image data is not great. Some of this can be mitigated by using recently added flow3D_smooth (see more information [here](https://cellpose.readthedocs.io/en/latest/do3d.html#segmentation-settings)), but we find the convex hull method to work quite well. The only inputs to create the convex hull image are the Solidity, BoundingBox, and ConvexImage:

B = regionprops3(A, "Solidity", "BoundingBox", "ConvexImage");

% Calculate Convex region properties

[A_Convex] = CreateConvexHullImage(BaseFileName, A, B, 1);

B_Convex = regionprops3(A_Convex,RawImage, "Centroid", "Volume", "MeanIntensity", "PrincipalAxisLength", "ConvexHull", "SurfaceArea");

To save the convex hull image for future use, or reading into ImageJ, we save as and HDF5 file:

h5create(FileName +"A_Convex.h5","/DS1",size(A_Convex))

h5write(FileName +"A_Convex.h5","/DS1",A_Convex)

or if the convex hull HDF5 file was already created and needs to be reloaded:

h5disp(FileName +"A_Convex.h5","/DS1")

A_Convex = h5read(FileName +"A_Convex.h5","/DS1");

**Create 3D volumetric arrays of cellular properties.**

Next, the information calculated by regionprops3 is converted into a 3D array with the same dimensions as the input image file. Thus far, we have written the functions to create image arrays of:

- Cell volume (VolumeCalculation.m)
- Staining marker concentration per cell (ActinConcentration.m)
- The ratio of the major and minor axes of each cell (MajorMinorRatio.m)
- The cell length, or longest principle axis length of the cell (CellLength.m)
- The 3D cell sphericity (SphericityCalculation.m)
- And the cell density (CellDensityCalculation.m)

As an example, we can create a 3D array of the cell volume using:

[A_vol] = VolumeCalculation(FileName, A, B, WriteTiff)

Where FileName is the base file name with path included (this is used when writing a TIF output), A is the segmented image, B is the output from regionprops3 as shown above, and WriteTiff is a Boolean function that instructs the function to write the array to a tiff file or not (1=True, anything else won't). This can also read the convex hull version as well:

[A_vol_Convex] = VolumeCalculation(FileName, A_Convex, B_Convex, 1);

For the sphericity calculation, there are instead 4 outputs:

- B_Sphericity_array is a list of the indexed cell sphericities simply using the "Volume" and "SurfaceArea" outputs from regionprops3. If the input segments are directly from cellpose and not smoothed using the convex hull approach described above, we find this data to be very noisy and not very useful.
- B_Sphericity_array_Ellipsoid is a list of the indexed cell sphericities calculated by approximating the surface area and volume of the cell using the three principle axes calculated by regionprops3. This assumes that every cell is essentially ellipsoidal in shape. While not perfect, it does circumvent the issue of fragmented cell segments output from Cellpose. We find the data to look very good and is quite representative.
- A_Sphericity is the output 3D image array of the B_Sphericity_array data.
- A_Sphericity_Ellipsoid is the output 3D image array of the B_Sphericity_array_Ellipsoid data.

**Data saving**

While Tiff files are a decent way to save data for visualization, the data exists as integers, which is not well suited for scientific data. Instead saving the data directly as an HDF5 file is best for preserving double-precision numerical data arrays:

h5create(FileName +"A_vol_Convex.h5","/DS1",size(A_vol_Convex))

h5write(FileName +"A_vol_Convex.h5","/DS1",A_vol_Convex)

For visualization in presentations, sometimes it's better to save GIF files:

%% Output as Gif

s = sliceViewer(A_vol,"Parent",figure,"Colormap",parula, "DisplayRange",[P_vol(1) P_vol(5)])

colorbar

hAx = getAxesHandle(s);

filenamegif = FileName + "_Volume.gif"

sliceNums = 1:size(A,3);

for idx = sliceNums

% Update slice number

s.SliceNumber = idx;

% Use getframe to capture image

I = getframe(hAx);

[indI,cm] = rgb2ind(I.cdata,256);

% Write frame to the GIF file

if idx == 1

imwrite(indI,cm,filenamegif,"gif","Loopcount",inf,"DelayTime", 0.01);

else

imwrite(indI,cm,filenamegif,"gif","WriteMode","append","DelayTime", 0.01);

end

end
